# DIS3 ribonuclease is essential for spermatogenesis and male fertility in mice

**DOI:** 10.1242/dev.202579

**Published:** 2024-07-02

**Authors:** Zhengpin Wang, Di Wu, Xiaojiang Xu, Guoyun Yu, Nana Li, Xiao Wang, Jian-Liang Li, Jurrien Dean

**Affiliations:** ^1^ Laboratory of Cellular and Developmental Biology, NIDDK, National Institutes of Health, Bethesda, MD 20892, USA; ^2^ Shandong Provincial Key Laboratory of Animal Cell and Developmental Biology, School of Life Sciences, Shandong University, Qingdao 266237, China; ^3^ Integrative Bioinformatics Support Group, NIEHS, National Institutes of Health, Research Triangle Park, NC 27709, USA

**Keywords:** DIS3, Spermatogenesis, Male fertility

## Abstract

Spermatogonial stem cell (SSC) self-renewal and differentiation provide foundational support for long-term, steady-state spermatogenesis in mammals. Here, we have investigated the essential role of RNA exosome associated DIS3 ribonuclease in maintaining spermatogonial homeostasis and facilitating germ cell differentiation. We have established male germ-cell *Dis3* conditional knockout (cKO) mice in which the first and subsequent waves of spermatogenesis are disrupted. This leads to a Sertoli cell-only phenotype and sterility in adult male mice. Bulk RNA-seq documents that *Dis3* deficiency partially abolishes RNA degradation and causes significant increases in the abundance of transcripts. This also includes pervasively transcribed PROMoter uPstream Transcripts (PROMPTs), which accumulate robustly in *Dis3* cKO testes. In addition, scRNA-seq analysis indicates that *Dis3* deficiency in spermatogonia significantly disrupts RNA metabolism and gene expression, and impairs early germline cell development. Overall, we document that exosome-associated DIS3 ribonuclease plays crucial roles in maintaining early male germ cell lineage in mice.

## Teaser

**Summary:** Conditional disruption of DIS3 causes severe impairment of early germline cell differentiation and subsequent failure of spermatogenesis through perturbation of RNA metabolism in male germ cells.

## INTRODUCTION

Mammalian spermatogenesis is a dynamic and coordinated process of cell differentiation that is sustained by a balance between maintaining a population of self-renewing spermatogonial stem cells (SSCs) versus their amplification and development into mature haploid spermatozoa. Abnormalities in this equilibrium either impair spermatogenic lineage development or lead to germline loss, which precludes spermatogenesis and male fertility (Yang and Oatley, 2014). In mice, SSCs are derived from prospermatogonial precursors (known as gonocytes) that arise from primordial germ cells formed in the proximal epiblast during embryogenesis (McCarrey, 2013; McLaren, 2000; Richardson and Lehmann, 2010). Prospermatogonia proliferate before entering cell-cycle arrest at embryonic day 16.5 (E16.5) and resume mitosis after birth to generate spermatogonia in mice (Hilscher et al., 1974; Kluin and de Rooij, 1981; Nagano et al., 2000). Post-natal SSCs are located at the basement membrane of seminiferous tubules and represent a small subset of the undifferentiated spermatogonial population. SSCs initiate mitotic divisions either to produce two new A_single_ (A_s_) spermatogonia by complete cytokinesis or to generate chains of A_paired_ (A_pr_) and A_aligned_ [A_al(4)_, A_al(8)_, A_al(16)_, and in rare cases, A_al(32)_] spermatogonia that are connected by intercellular bridges due to incomplete cytokinesis. A_s_, A_pr_ and A_al_ cells represent undifferentiated spermatogonia. Progenitor spermatogonia have a limited capacity to proliferate before transitioning to A_1_ spermatogonia, which undergo a series of mitotic divisions to sequentially form A_2_, A_3_, A_4_, intermediate (In) and type B spermatogonia that are collectively termed differentiated spermatogonia (de Rooij, 2001; Phillips et al., 2010; Song and Wilkinson, 2014). Type B spermatogonia give rise to two primary spermatocytes that meiotically divide twice to produce haploid round spermatids that initiate spermiogenesis and form mature spermatozoa (Fayomi and Orwig, 2018).

Considerable investigative effort has been devoted to discovering developmentally regulated transcriptional networks required for mouse spermatogenesis (Green et al., 2018; Hermann et al., 2018; Shami et al., 2020; Tan et al., 2020). However, once a particular developmental stage has passed, it is necessary to reconfigure the transcriptome for the next step of spermatogenesis both by *de novo* transcription and by degradation of no longer needed RNAs. Multiple RNases and co-factors have evolved in mice to ensure a developmentally correct transcriptome (Jamin et al., 2017; Morgan et al., 2019; Suzuki et al., 2016; Yamaji et al., 2017). One example is the RNA exosome, which is a multi-protein complex composed of nine subunits that form a two-layered barrel-like structure, arranged as a core ring structure of six conserved proteins and capped by three RNA-binding proteins (Symmons and Luisi, 2009). The RNA exosome core complex lacks catalytic activity, which is conferred by one of three catalytic ribonucleases: EXOSC10 (yeast homologue, RRP6), DIS3 (RRP44) or DIS3L (Makino et al., 2013). EXOSC10 is expressed in the nucleus and enriched within the nucleolus (Tomecki et al., 2010). DIS3 enzyme also exists primarily in the nucleus, whereas DIS3L is strictly cytoplasmic (Robinson et al., 2015; Tomecki et al., 2010). The DIS3 enzyme belongs to RNase II/R family and encodes a highly conserved ribonuclease that possesses both 3′ to 5′ exoribonuclease and endoribonuclease activities (Dziembowski et al., 2007; Lebreton et al., 2008; Schaeffer et al., 2009).

Genetic mutations of exosome-related genes and disorders of RNA degradation have been linked to a range of human diseases (Robinson et al., 2015; Saramago et al., 2019). Disruption of DIS3 ribonuclease is associated with human cancers, including melanoma, multiple myeloma and leukemias (Ng et al., 2007; Weißbach et al., 2015). Perturbation of RNA degradation in *Exosc10* conditional null oocytes results in disruption of meiotic resumption and failure of pre-implantation embryo development in mice (Wu and Dean, 2020). EXOSC10 is associated with epigenetic chromosome silencing and is essential for mouse male germ cell proliferation and development (Jamin et al., 2017). However, genetic models of exosome-associated disease are still lacking and whether DIS3-associated RNA metabolism is linked with reproductive abnormalities remains unknown.

In this study, we determined an essential role for exosome-associated DIS3 ribonuclease in maintaining spermatogonial homeostasis. We have established *Dis3* conditional knockout mice and the loss of DIS3 in male germ cells severely impairs early germline cell development. This leads to defects in spermatogenesis and results in a Sertoli cell-only phenotype of adult sterility. RNA-seq analysis indicates that the absence of DIS3 causes significant dysregulation of transcripts and accumulation of PROMPTs. scRNA-seq analysis further documents that abnormal RNA degradation resulting from *Dis3* ablation impairs spermatogonial development. Thus, our findings conclusively indicate that exosome-associated DIS3 ribonuclease is essential for early spermatogenic maintenance in mice.

## RESULTS

### *Dis3* expression in the mouse testes

**Fig. 1.**
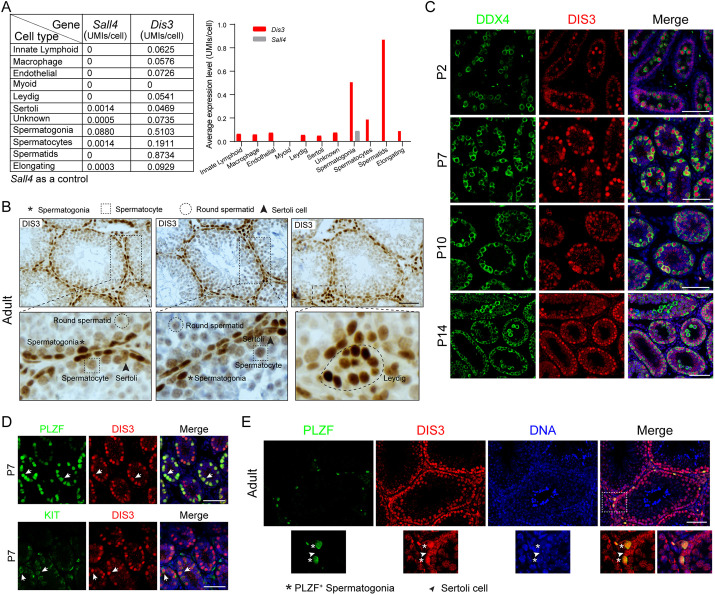
**The expression of DIS3 in wild-type testes.** (A) Analysis of the abundance of *Dis3* transcripts in 11 testicular cell types based on previously reported scRNA-seq data in adult mouse testes (Green et al., 2018). Numbers in the table (left) and on the *y*-axis (right) show the averaged UMIs detected in each individual cell. *Sall4* expression is used as a control. (B) Immunohistochemistry of DIS3 in adult testes. Asterisks indicate spermatogonia. Dashed rectangles indicate spermatocytes. Dashed circles indicate round spermatids. Arrowheads indicate Sertoli cells. Scale bar: 50 µm. (C) Immunofluorescence of cross-sections from P2, P7, P10 and P14 wild-type testes after staining with antibodies to DDX4 (left) or DIS3 (middle), and merged with Hoechst 33342 to stain DNA (right). Scale bars: 50 µm. (D) Immunofluorescence of cross-sections from P7 wild-type testes after staining using antibodies to PLZF and DIS3 (top), KIT and DIS3 (bottom). Arrows indicate both PLZF- and DIS3-positive cells (top), and KIT- and DIS3-positive cells (bottom). (E) Immunofluorescence of cross-sections from adult wild-type testes after staining using antibodies to PLZF and DIS3. Asterisks indicate PLZF-positive spermatogonia. Arrowheads indicate Sertoli cells. Images are representative of three samples. (B-E) Independent biological replicates with similar results per condition.

Using reported scRNA-seq data (Green et al., 2018), we determined *Dis3* expression in adult mouse testes and found that *Dis3* transcripts were ubiquitously expressed in both germ and somatic cells (Fig. 1A). To characterize the DIS3 protein expression in adult testes, we examined its accumulation by immunohistochemistry with an antibody to the ribonuclease. The data documented that DIS3 was expressed in the nucleus of spermatogonia, spermatocytes, round spermatids, Sertoli cells and Leydig cells (Fig. 1B). Moreover, we characterized DIS3 expression in post-natal testes from P2 to P14 during early male germ cell development, and demonstrated that DIS3 was expressed in the nucleus of spermatogonia in P2, P7, P10 and P14 wild-type testes, as indicated by co-staining with germ cell-specific marker DDX4 (Fig. 1C). Co-expression of DIS3 with either PLZF or KIT in P7 and adult testes indicated that DIS3 ribonuclease was present in both undifferentiated and differentiated spermatogonia (Fig. 1D,E). These findings demonstrate the nuclear presence of DIS3 ribonuclease in spermatogonia and provide a rationale for conditional inactivation of *Dis3* in spermatogonia to elucidate the function of DIS3 in early male germline development.

### DIS3 is essential for maintenance of spermatogenesis and male fertility

**Fig. 2.**
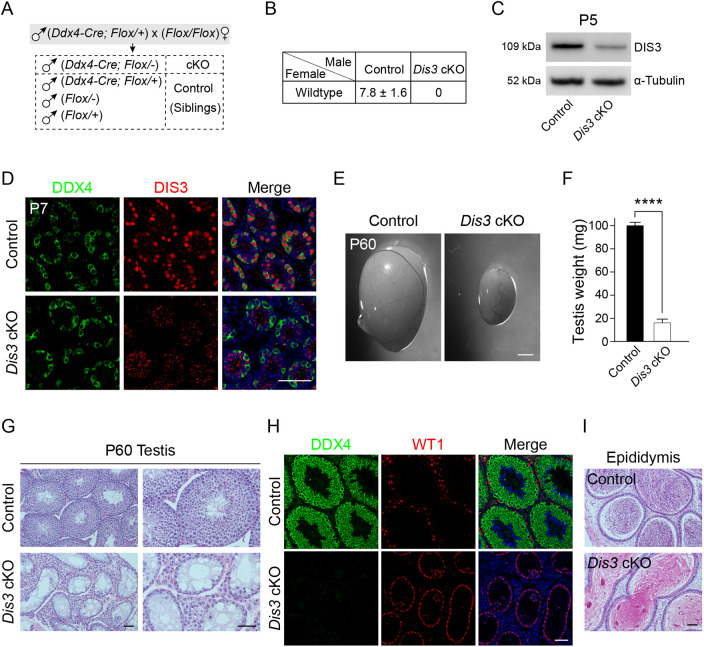
***Dis3* is required for spermatogenesis and male fertility.** (A) Schematic of mating strategies to generate *Dis3* cKO male mice. (B) Fertility of control and *Dis3* cKO male mice mated 1:1 with wild-type female mice. Data are mean litter size±s.d. (C) Immunoblot assay of DIS3 protein in P5 control and *Dis3* cKO testes using α-tubulin as a loading control. (D) Immunofluorescence of cross-sections from P7 control and *Dis3* cKO testes after staining using antibodies to DDX4 (left) or DIS3 (middle), and merged with Hoechst 33342 to stain DNA (right). Scale bar: 50 µm. (E) Representative morphology of testes from P60 control and *Dis3* cKO mice. Scale bar: 1 mm. (F) Testis weight of P60 control and *Dis3* cKO mice. Data are mean±s.d., *n*=4 biologically independent testes from four different animals. ****P<0.0001. (G) Adult testicular sections from control and *Dis3* cKO mice stained with periodic acid-Schiff (PAS) and Hematoxylin. Right column shows magnification of images in left column. Scale bars: 50 µm. (H) Immunofluorescence of P60 adult testes from control and *Dis3* cKO mice after co-staining using antibodies to DDX4 (germ cells) and WT1 (Sertoli cells), as well as Hoechst 33342 (DNA). Scale bar: 50 µm. (I) Cauda epididymides sections from control and *Dis3* cKO mice stained with periodic acid-Schiff (PAS) and Hematoxylin. Scale bar: 50 µm. Images are representative of three samples. (C,D,G-I) Independent biological replicates with similar results per condition.

To investigate the function of DIS3 ribonuclease in early male germline development, we used previously established *Dis3^Floxed/Floxed^* (*Dis3^F/F^*) mice (Fig. S1A) (Wu and Dean, 2023). The floxed mice were crossed with *Ddx4-Cre* transgenic mice (Fig. 2A, Fig. S1B) in which the recombinase is expressed in male germ cells as early as at E15.5 (Gallardo et al., 2007). In the resultant conditional mutant mice, exons 4 to 6 of *Dis3* were deleted and a frameshift mutation was introduced in male germ cells. Immunoblots documented significant reduction of DIS3 protein abundance in post-natal day 5 (P5) testes of *Dis3^F/−^*; *Ddx4-Cre* mice (referred to as *Dis3* cKO) compared with controls (siblings in the same litter) (Fig. 2C). Immunohistochemistry determined that DIS3 was completely absent in DDX4-positive germ cells in P7 *Dis3* cKO testes (Fig. 2D). Thus, we successfully established a conditional knockout mouse line in which *Dis3* was specifically ablated in male germ cells.

To assess male fertility, control or *Dis3* cKO males were mated with wild-type females for 6 months and *Dis3* cKO males never produced pups (Fig. 2B). Testes isolated from 2-month-old *Dis3* cKO mice were significantly smaller and weighed less than control testes (Fig. 2E,F), which suggested abnormal spermatogenesis. Histologically, *Dis3* cKO seminiferous tubules lacked spermatogenic cells and contained only somatic cells, which defined a Sertoli cell-only phenotype (Fig. 2G). The absence of germ cells was confirmed by immunofluorescence staining in which the germ cell-specific marker DDX4 was not detected, but Wilms tumor 1 (WT1), a Sertoli cell-specific marker, was observed (Fig. 2H). Consistently, mature sperm were present in control but not *Dis3* cKO cauda epididymides (Fig. 2I). Thus, *Dis3* ablation in germ cells disrupts spermatogenesis, resulting in agametic seminiferous tubules in adult testes and male infertility.

### *Dis3* ablation impairs spermatogenic lineage development

**Fig. 3.**
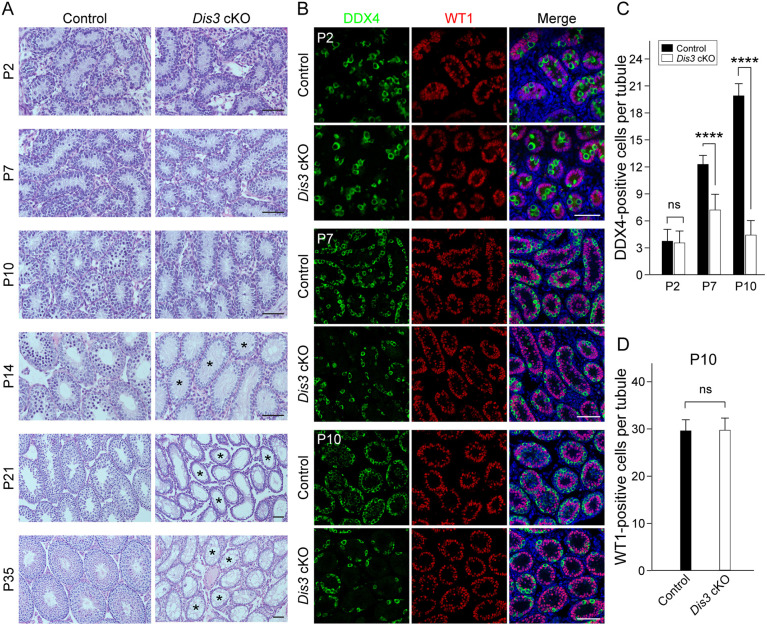
***Dis3* is required for early spermatogenic maintenance.** (A) Histological analysis of testicular sections from control and *Dis3* cKO mice stained with periodic acid-Schiff (PAS) and Hematoxylin at the indicated ages from P2 to P35. Asterisks indicate agametic tubules. Scale bars: 50 µm. (B) Immunofluorescence of sections from P2, P7 and P10 control and *Dis3* cKO testes after staining using antibodies to DDX4 (left) or WT1 (middle), and merged with Hoechst 33342 to stain DNA (right). Scale bars: 50 µm. (C) Statistical analysis of DDX4-positive cells per tubule in control and *Dis3* cKO testes at P2, P7 and P10. Data are mean±s.d., *n*=3 biologically independent testes from three different animals. ****P<0.0001. (D) Statistical analysis of WT1-positive cells per tubule in P10 control and *Dis3* cKO testes. Data are mean±s.d. Images are representative of three samples. (A,B) Independent biological replicates with similar results per condition.

To determine the stage at which defects in spermatogenesis occurred, morphological differences were analyzed in control and *Dis3* cKO testes. Histological and immunohistochemical analyses with DDX4 and WT1 staining documented that *Dis3* cKO testes and controls had a similar number of germ cells at P2 (Fig. 3A-C), suggesting that *Dis3* ablation has no phenotype before P2 in mutant testes. However, the number of germ cells was significantly reduced by P7 in mutant testes (Fig. 3A-C). The defects were more severe and notable by P10, when *Dis3* cKO tubules contained substantially fewer germ cells (Fig. 3A-C). Virtually no germ cells were present in P14 *Dis3* cKO tubules, and mutant seminiferous tubules were completely devoid of germ cells and lined only with Sertoli cells in P21 testes, as determined by immunohistochemistry (Fig. 3A, Fig. S1C,D). At P35, the first wave of spermatogenesis was complete in control testes and mature sperm were present in the epididymides. In contrast, *Dis3* cKO seminiferous tubules lacked spermatogenic cells and contained mostly Sertoli cells, and no mature spermatozoa were observed in the epididymides (Fig. 3A, Fig. S1E,F). However, the number of Sertoli cells in *Dis3* cKO testes was similar to control testes in P10 mice (Fig. 3D). Taken together, these observations suggest that conditional *Dis3* ablation in male germ cells causes rapid and profound depletion of spermatogonia and spermatogenic cells. This disrupts the first wave of spermatogenesis, and results in agametic seminiferous tubules and a Sertoli cell-only phenotype. Thus, DIS3 ribonuclease is required for the maintenance of early spermatogenic lineage in juvenile mouse testes.

### DIS3 ribonuclease is required for spermatogonial maintenance

**Fig. 4.**
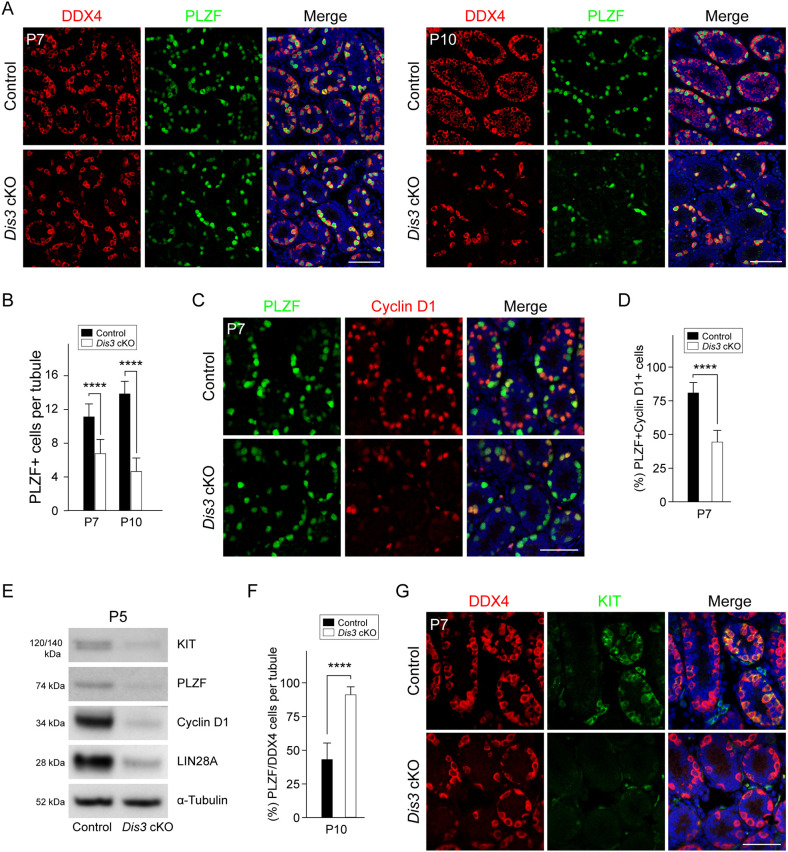
***Dis3* is indispensable for spermatogonial homeostasis.** (A) Immunofluorescence staining of control and *Dis3* cKO testes at P7 and P10 with antibodies to DDX4 (left) or PLZF (middle), and merged with Hoechst 33342 to stain DNA (right). Scale bars: 50 µm. (B) Quantification of PLZF-positive cells per tubule. Data are mean±s.d., ****P<0.0001. (C) Immunofluorescence staining of control and *Dis3* cKO testes at P7 and P10 using antibodies to PLZF and cyclin D1 at P7. (D) The ratio of PLZF- and cyclin D1-positive cells among the total of PLZF-positive cells. Data are mean±s.d., ****P<0.0001. (E) Immunoblot analyses of KIT, PLZF, cyclin D1 and LIN28A expression in P5 control and *Dis3* cKO testes using α-tubulin, as also shown in Fig. 2C, as a loading control. (F) Percentage of PLZF-positive cells to DDX4-positive cells per tubule in P10 control and *Dis3* cKO testes. Data are mean±s.d., ****P<0.0001. (G) Immunofluorescence staining of control and *Dis3* cKO testes at P7 and P10 using antibodies to DDX4 and KIT at P7. Images are representative of three samples. (A,C,E,G) Independent biological replicates with similar results per condition. (B,D,F) Biologically independent testes from three different animals were analyzed.

Compared with control mice, *Dis3* ablation results in similar number of germ cells at P2 but significantly reduced number of germ cells at P7, implying that *Dis3* deficiency impairs spermatogonial proliferation and expansion. Thus, we determined the overall number of germ cells and undifferentiated spermatogonia by whole-mount and immunofluorescence staining using antibodies to DDX4 and to promyelocytic leukemia zinc finger, PLZF (official name ZBTB16, a marker for undifferentiated spermatogonia) (Buaas et al., 2004). The number of DDX4-positive germ cells and PLZF-positive spermatogonia were comparable with controls at P2 but were substantially reduced in *Dis3* cKO tubules by P7 and, more severely, by P10 (Fig. 4A,B, Fig. S2A,B), suggesting a specific requirement for DIS3 ribonuclease in spermatogonial maintenance.

Next, we investigated spermatogonial proliferation in *Dis3* cKO tubules. Immunofluorescence staining with antibodies to PLZF and cyclin D1 (a marker for mitotically active spermatogonia) (Costoya et al., 2004) documented that the number of PLZF^+^; cyclin D1^+^ spermatogonia per tubular cross-section was dramatically decreased in P7 *Dis3* cKO testes compared with controls (Fig. 4C,D). Immunoblots documented that expression of PLZF, cyclin D1 and LIN28A (a marker for undifferentiated spermatogonia) was significantly decreased as early as P5 in *Dis3* cKO testes (Fig. 4E). Thus, we conclude that the proliferative capacity of spermatogonia is impaired in *Dis3* cKO testes. Moreover, co-staining of PLZF and cleaved caspase 3 indicated that the number of apoptotic spermatogonia in *Dis3* cKO tubules was not significantly different from controls (Fig. S2C,D). This was confirmed by immunoblot, which documented similar abundance of cleaved caspase 3 in control and *Dis3* cKO testes (Fig. S2E), and suggests that the observed impairment of spermatogonial expansion was not due to increased cell death. Taken together, these data indicate that *Dis3* ribonuclease deficiency leads to defects in spermatogonial proliferation and expansion that normally occur in early post-natal testes.

Notably, we found that the great majority of DDX4-positive germ cells in *Dis3* cKO tubules colocalized with PLZF in both P7 and P10 testes (Fig. 4A), indicating that undifferentiated spermatogonia were dominant in *Dis3* cKO testes. Statistical analysis demonstrated that ∼90% spermatogonia were PLZF positive in P10 *Dis3* cKO testes (Fig. 4F), implying impairment of spermatogonial differentiation. To determine the role of DIS3 ribonuclease in spermatogonial differentiation, we assayed for co-expression of DDX4 and KIT (a marker for differentiated spermatogonia). KIT-positive spermatogonia were barely detected in P7 *Dis3* cKO testes, whereas a population of KIT-positive spermatogonia was present at the same time point in control testes (Fig. 4G). This observation was further confirmed by immunoblots that documented that KIT protein was significantly reduced as early as P5 in *Dis3* cKO testes (Fig. 4E). Additionally, γH2AX-positive cells were rarely observed in *Dis3* cKO testes at P10, which suggests that very few differentiated spermatogonia were present and able to enter meiosis in *Dis3* cKO testes (Fig. S2F). These results indicate that DIS3 ribonuclease is required for spermatogonial maintenance. Disruption of *Dis3* impairs early spermatogenic maintenance and causes a dramatic decline in undifferentiated and differentiated spermatogonial populations.

### DIS3 ribonuclease is required for GFRA1^+^ undifferentiated spermatogonia

**Fig. 5.**
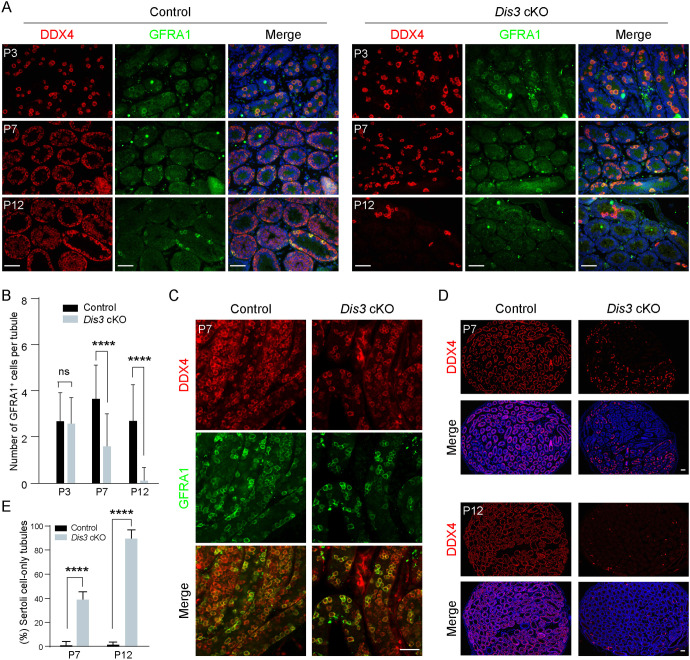
**DIS3 is required for maintenance of GFRA1^+^ undifferentiated spermatogonia.** (A) Immunofluorescence of cross-sections from P3, P7 and P12 control and *Dis3* cKO testes after staining with antibodies to DDX4 or GFRA1, and merged with Hoechst 33342 to stain DNA. Scale bars: 50 µm. (B) Statistical analysis of GFRA1-positive cells between control and *Dis3* cKO testes at P3, P7 and P12. Data are mean±s.d., ns, no significance; ****P<0.0001. (C) Whole-mount staining of testes from P7 control and *Dis3* cKO mice after staining using antibodies to DDX4 and GFRA1. Scale bar: 50 µm. (D) Immunostaining of DDX4 in P7 and P12 control and *Dis3* cKO testes. Scale bars: 50 µm. (E) The percentage of Sertoli cell-only tubules in P7 and P12 control and *Dis3* cKO testes. Data are mean±s.d., ****P<0.0001. (A,C,D) Images are representative of three independent biological replicates with similar results per condition.

To determine whether DIS3 affects the maintenance of GFRA1^+^ undifferentiated spermatogonia, we conducted co-staining of GFRA1 and DDX4 in control and *Dis3* cKO testicular sections at P3, P7 and P12. The results indicated that the number of GFRA1-positive spermatogonia was comparable in control and *Dis3* cKO testes at P3 (Fig. 5A,B). However, the number was substantially reduced in *Dis3* cKO testes by P7 (Fig. 5A,B), which was also confirmed by whole-mount staining with antibodies to DDX4 and GFRA1 (Fig. 5C). The GFRA1-positive spermatogonia were rarely observed in *Dis3* cKO testes at P12, as germ cells were almost absent in the testes (Fig. 5A,B). Moreover, we analyzed the percentage of Sertoli cell-only tubules in P7 and P12 control and *Dis3* cKO testes. The data showed that ∼40% of the tubules were devoid of germ cells and lined only with Sertoli cells in P7 *Dis3* cKO testes. Over 90% of the tubules displayed Sertoli cell-only phenotype and the remaining tubules contained very few DDX4-positive germ cells in P12 *Dis3* cKO testes (Fig. 5D,E). Collectively, these results demonstrate that DIS3 is required for maintenance of GFRA1^+^ undifferentiated spermatogonia, which are rapidly lost and disappeared in the testes as early as at P12 after DIS3 deletion.

### DIS3 ribonuclease ablation in male germ cells causes dysregulation of transcripts

**Fig. 6.**
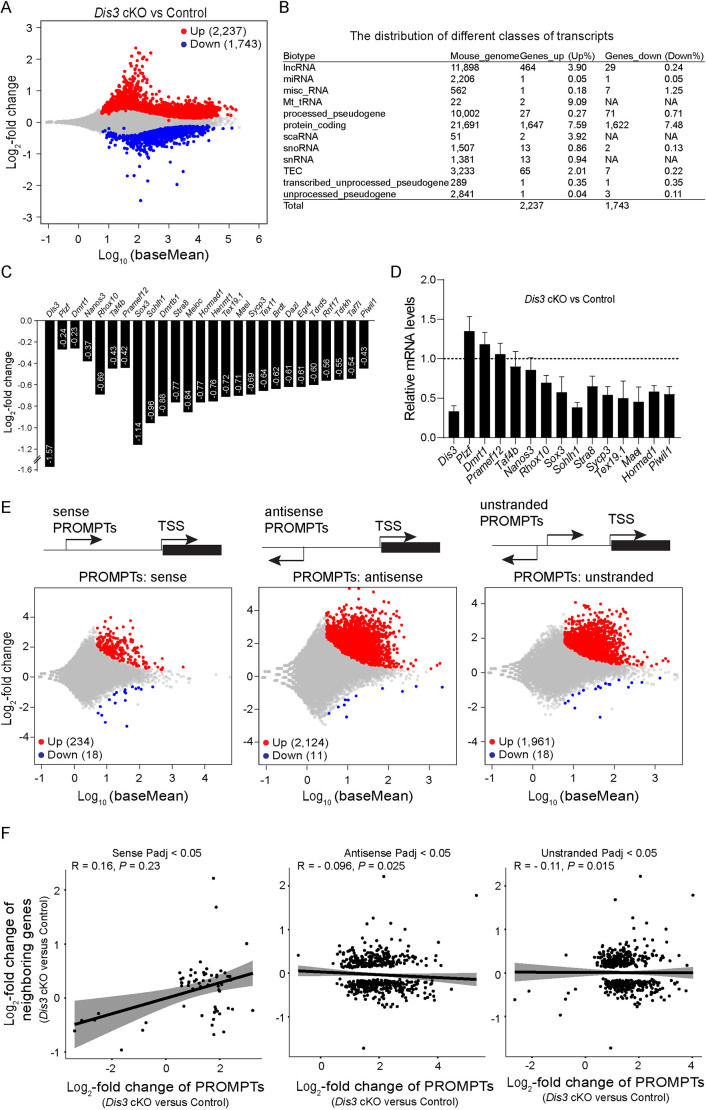
**Significant dysregulation of the transcriptome caused by DIS3 ablation.** (A) MA plot (log ratio RNA abundance versus abundance) of RNA-seq data from control and *Dis3* cKO testes at P4, using adjusted *P*<0.05 as the cut off. (B) The distribution of the up- and downregulated genes over different classes of transcripts. (C) RNA-seq results of selected transcripts (log_2_-fold change) related to spermatogonial development and spermatogenesis in *Dis3* cKO testes. (D) Quantitative RT-PCR validation of downregulated genes involved in spermatogonial development and spermatogenesis. For comparison, the abundance (relative to β-actin) of each gene in control testes was set to 1. Data are mean±s.d. for *n*=3 biologically independent samples per condition. (E) PROMPTs analysis represented as MA plots (log ratio RNA abundance versus abundance) of RNA-seq data from *Dis3* cKO and control testes using an adjusted *P*<0.05 as the cut off. Sequence reads were mapped to the PROMPTs regions of the mouse genome and aligned with the sense strand (left panel), antisense strand (middle panel) and in an unstranded manner (right panel) for PROMPTs analysis. (F) Correlation analysis between the differentially expressed PROMPTs and the expression of their neighboring genes. The relationship of expression fold changes between control and *Dis3* mutant for PROMPTs and their neighboring transcripts, as shown by Pearson correlation coefficient analysis.

To investigate the molecular consequences of *Dis3* ablation in early post-natal germline cell development, we performed bulk RNA-sequencing (RNA-seq) and compared the transcriptome at P4 between control and *Dis3* cKO testes. RNA-seq results documented that 2237 transcripts were significantly upregulated and 1743 transcripts were downregulated in *Dis3* cKO testes using an adjusted *P*<0.05 (Fig. 6A). We divided the transcripts into different biotypes to identify testicular targets of DIS3 ribonuclease. Importantly, we observed that lncRNA, snRNA, snoRNA and protein-coding genes were the primary biotypes of the upregulated transcripts in the absence of DIS3 ribonuclease in testes, whereas lncRNA, processed pseudogene and protein-coding genes were the main biotypes of downregulated transcripts upon the loss of DIS3 ribonuclease (Fig. 6B).

RNA-seq analysis suggested that genes related to spermatogonial development and spermatogenesis were dramatically reduced in *Dis3* cKO testes (Fig. 6C). The qRT-PCR results confirmed the significant downregulation of several genes (e.g. *Rhox10*, *Sox3*, *Sohlh1*, *Stra8*, *Sycp3*, *Tex19.1*, *Mael*, *Hormad1* and *Piwil1*) involved in spermatogonial development and spermatogenesis (Fig. 6D). However, the downregulation of these spermatogenic genes is most likely a secondary effect caused by loss of DIS3 ribonuclease, as will be indicated in the Discussion. Collectively, these data suggest that germ cell-specific ablation of DIS3 ribonuclease leads to significant disruption of transcripts in post-natal testes.

Gene Ontology (GO) analysis documented that the transcripts with increased abundance were primarily involved in regulation of transcription, cell adhesion and cell migration (Fig. S3A). Kyoto Encyclopedia of Genes and Genomes (KEGG) pathway analysis documented that the upregulated transcripts were enriched in GnRH signaling pathway (Fig. S3B). In addition, downregulated transcripts were significantly enriched in GO terms of biological processes, including ‘cell cycle’, ‘DNA replication’, ‘spermatogenesis’ and ‘RNA processing’ (Fig. S3C), indicative of impairment of cell proliferation after *Dis3* ablation. In addition, KEGG analysis of downregulated genes indicated that genes associated with the terms ‘cell cycle’, ‘DNA replication’ and ‘mRNA surveillance pathway’ were severely disrupted by DIS3 ribonuclease inactivation (Fig. S3D).

### DIS3 inactivation leads to accumulation of PROMPTs

PROMPTs are produced upstream of active transcription start sites. They share characteristics with mRNAs and can be transcribed bidirectionally with respect to the downstream genes (Preker et al., 2011, 2008). To obtain a general overview of the impact of DIS3 ablation in male germ cells on PROMPTs, we defined the PROMPT region and prepared the PROMPT reference file for subsequent analyses. Sequencing reads were mapped to −3 kb to −1 bp upstream of the transcription start site of a subset of 30,166 genes from the current mouse genome that were selected not to overlap with any other annotated gene feature. Based on the characteristic of the bi-directional transcription activity of PROMPTs, we aligned the sequencing reads in the direction of sense, antisense or both (unstranded) to the PROMPT reference file. RNA-seq comparison of *Dis3* cKO and control testes documented that 234 transcript-associated PROMPTs were increased in the sense direction, 2124 PROMPTs were increased in the reverse direction and 1961 PROMPTs were unstranded (Fig. 6E). Thus, we observed that PROMPTs primarily accumulated in the reverse direction, which was consistent with the previous findings that PROMPT transcription generally initiates in the antisense direction with respect to downstream genes (Lloret-Llinares et al., 2016; Ntini et al., 2013). To define the RNA targets, we examined the distribution of downstream genes over different classes of transcripts. LncRNA, processed pseudogene, snoRNA, snRNA and protein-coding genes were the primary biotypes affected by the loss of DIS3 ribonuclease (Fig. S3E), consistent with the effect of DIS3 on the whole transcriptome. Next, we determined whether the differentially expressed PROMPTs were correlated with the expression of their neighboring genes. Despite robust accumulation of PROMPTs, we observed no positive or only very weak negative correlation with expression of adjacent genes by Pearson correlation coefficient analysis (Fig. 6F).

### scRNA-seq defines the transcriptome of *Dis3* cKO testes

To address the spermatogonia composition and transcriptome changes in *Dis3* cKO testes, we isolated single cells from P6 control and *Dis3* cKO testes, and performed scRNA-seq analysis of the testicular cells using the 10X Genomics platform. From a total number of 17,242 control and 12,794 *Dis3* cKO testicular cells, 16,429 and 12,015 cells passed the standard quality control, respectively, and were retained for subsequent analysis (Fig. S4A). We detected an average of 16,029 UMIs and 3830 genes in each individual cell in control testes, and an average of 18,051 UMIs and 4205 genes in *Dis3* cKO testes (Fig. S4A). The sequencing depth is sufficient for defining distinct cell types and for differential gene expression analysis in mouse testis. Uniform Manifold Approximation and Projection (UMAP) and marker gene analyses were performed for cell type identification of the combined, control and *Dis3* cKO testicular cells. We identified seven cell types based on expression patterns of known marker genes in mouse testis, including a germ cell population and six somatic cell populations: endothelial cells, macrophages, myoid cells, Leydig cells, stroma and Sertoli cells (Fig. S4B,C).

### Transcriptome-wide signatures of *Dis3* cKO spermatogonia

**Fig. 7.**
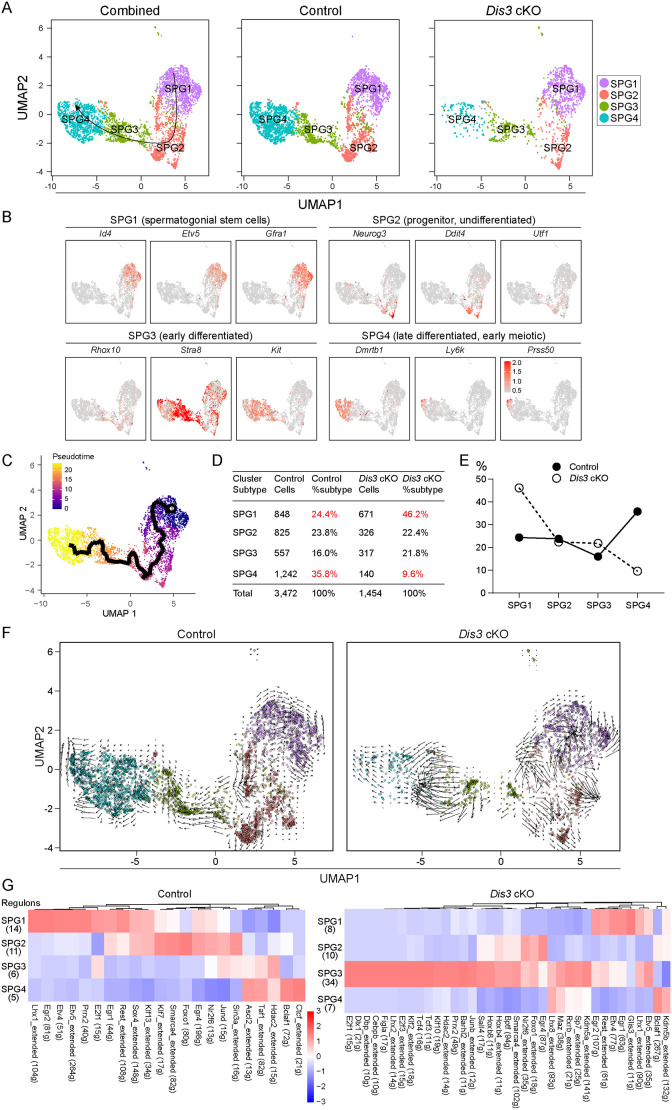
**scRNA-seq analysis of transcriptome signatures of *Dis3* cKO spermatogonia.** (A) UMAP plots of combined (left panel), control (middle panel) and *Dis3* cKO (right panel) spermatogonia had four distinct spermatogonial subtypes following the developmental order of SPG1 to SPG4 (state 1 to state 4). (B) UMAP plots of selected marker genes corresponding to each cellular state. (C) Pseudotime trajectory of the combined four spermatogonial subtypes. (D) Summary of detailed cell numbers and percentages of spermatogonia in each cell cluster subtype in control and *Dis3* cKO testes. (E) The distribution of the percentages of spermatogonial subtypes in control and *Dis3* cKO samples. (F) RNA velocity dynamics in spermatogonial cell clusters in control and *Dis3* cKO samples. RNA velocities were visualized on existing embedding UMAP. (G) SCENIC analysis of regulons identified in spermatogonial cell clusters in control and *Dis3* cKO samples. Number of regulons and heatmap of regulon activity within each subtype in control (left panel) and *Dis3* cKO (right panel) samples are shown. The labels ‘104g’, ‘81g’, etc., in parentheses on the *x*-axis indicate the direct target gene numbers of the corresponding transcription factor.

To determine how DIS3 ribonuclease impacts cellular heterogeneity and transcriptome-wide signatures of spermatogonia during SSC development, we re-clustered germ cells from P6 testes. After filtering out cells of poor quality, 4956 spermatogonia were analyzed (Fig. S5A) and, based on UMAP and marker gene analyses, we identified four distinct spermatogonial subtypes (SPG1, SPG2, SPG3 and SPG4) (Fig. 7A, Table S1). Cluster identity was assigned based on expression of spermatogonial markers (Fig. 7B). SPG1 cells correspond to SSCs and expressed the highest levels of *Id4*, *Etv5* and *Gfra1*. SPG2 cells expressed high levels of *Neurog3*, *Ddit4* and *Utf1*, consistent with progenitor/undifferentiated spermatogonia. SPG3 cells expressed early differentiation marker genes, including *Rhox10*, *Stra8* and *Kit*, and SPG4 cells expressed *Dmrtb1*, *Ly6k* and *Prss50*, which identified them as late differentiated spermatogonia. Monocle pseudotime analysis provided a developmental trajectory of spermatogonial cells from SPG1 to SPG4 (Fig. 7C). Therefore, the expression pattern of spermatogonial marker genes and pseudotime analysis recapitulate the developmental order of germline cells from state 1 (SSCs) to state 4 (differentiated spermatogonia).

We determined that 24.4%, 23.8%, 16.0% and 35.8% cells were sorted into SPG1, SPG2, SPG3 and SPG4 subtypes, respectively, in control samples, and 46.2%, 22.4%, 21.8% and 9.6% cells were present in SPG1, SPG2, SPG3 and SPG4 subtypes, respectively, in *Dis3* cKO samples (Fig. 7D,E). We observed significant accumulation of cells in the SPG1 subtype and a dramatic decrease of cells in the SPG4 subtype of *Dis3* cKO samples. This indicates impairment of proliferation and differentiation that leads to accumulation of spermatogonial cells in SPG1 (SSCs) in the absence of DIS3 ribonuclease. Differential gene expression analyses were conducted for each subtype (Fig. S5B,C, Table S2) and Gene Set Enrichment Analysis (GSEA) determined the biological processes (GO terms) and pathways enriched in *Dis3* cKO cells for each subtype (Fig. S6A,B). In assessing DEG lists, volcano plots and bar graphs showed distinct differences in the DEGs in each subtype (Fig. S5B,C). Using a cutoff of *P*<0.05 and log_2_ fold-change >0.1 to detect subtle influences on the cell, 1927 DEGs (1387 upregulated and 540 downregulated) were identified in SPG1, 3017 DEGs (1996 upregulated and 1021 downregulated) were identified in SPG2, 3535 DEGs (2110 upregulated and 1425 downregulated) were identified in SPG3 and 3722 DEGs (2460 upregulated and 1262 downregulated) were identified in SPG4 (Fig. S5C, left panel). When restricted to a more stringent *P*<0.05 and log_2_ fold-change >0.2, these values were 557 (418 upregulated and 139 downregulated; SPG1), 1045 (754 upregulated and 291 downregulated; SPG2), 1078 (715 upregulated and 363 downregulated; SPG3) and 1429 (970 upregulated and 459 downregulated; SPG4) (Fig. S5C, right panel). It was apparent that the majority of genes were dysregulated and upregulated gene numbers were higher than downregulated genes in response to *Dis3* deletion in each subtype, suggesting defective RNA degradation. In assessing enriched GO terms of biological processes, terms associated with extracellular matrix and regulation of mRNA processing were significantly enriched, whereas GO terms associated with ribosome and RNA catabolic process were markedly decreased in the SPG1 subtype of *Dis3* cKO cells (Fig. S6A). Consistently, pathway enrichment analyses documented that genes related to extracellular matrix organization and HDACs deacetylate histones were enriched, whereas genes related to translation, metabolism of RNA, DNA replication and cell cycle were downregulated in *Dis3* cKO SPG1 cells (Fig. S6B). The patterns of GO terms and pathways in SPG2, SPG3 and SPG4 subtypes of *Dis3* cKO cells were very similar to SPG1 (Fig. S6A,B). Thus, single-cell transcriptional analysis of post-natal germline cells indicates that DIS3 ribonuclease plays a crucial role in germline cell development. The absence of DIS3 causes a defect in RNA degradation that perturbs the balance between RNA transcription and degradation, leading to disruption of RNA metabolism and gene expression. Taken together, these data demonstrate that loss of DIS3 ribonuclease significantly impairs early germline cell development, which eventually disrupts maintenance of early spermatogenesis.

### RNA velocity dynamics and SCENIC analysis of *Dis3* cKO spermatogonia

To extend these observations, we performed RNA velocity analysis in control and *Dis3* cKO spermatogonia. RNA velocity is the time derivative of the gene expression and can be determined by distinguishing unspliced and spliced mRNAs using common scRNA-seq protocols. RNA velocity predicts future transcriptional states of individual cells and, thus, infers transcriptional and developmental trajectories (La Manno et al., 2018). To show the validity of the RNA velocity analysis, we first determined whether *Dis3* cKO alters the ratios between spliced and unspliced transcripts. We employed the Picard tools CollectRnaSeqMetrics to produce a percentage of UTR, exon, intron and intergenic reads for RNA-seq data, and showed that there was no significant difference between control and *Dis3* cKO in terms of percentage of fragments mapped to various elements of genes (Table S3). RNA velocity dynamics recapitulated the developmental trajectory of germline cells from SPG1 to SPG4 in control testes (Fig. 7F), consistent with previous pseudotime analyses. We also noted that this analysis identified RNA velocity dynamics that exhibited a distinct SSC self-renewal feature within the SPG1 cluster, as well as an apparent and smooth progression pattern from SPG2 to SPG4 in control cells (Fig. 7F). In contrast, the transcriptional states of *Dis3* cKO SPG1 and SPG2 cells were extremely disorganized, and some cells in the SPG1 cluster had incoherent velocity vectors. Cells in the SPG2 cluster displayed long velocity vectors pointing in different directions, but never toward SPG3 cells (Fig. 7F), suggesting disrupted differentiation. This analysis implies that transcriptional and cell states were severely disrupted upon DIS3 ribonuclease depletion in early post-natal germline cells.

To further explore the transcriptional state of germ cells, we used SCENIC (single-cell regulatory network inference and clustering) analysis to reconstruct gene regulatory networks and define cell states from the scRNA-seq data (Aibar et al., 2017). Although DIS3 functions in RNA decay pathways, dysregulated downstream genes can affect transcription through DNA and histone modifications, and we observed significant enrichment of histone deacetylases (HDAC) and DNA methylases in *Dis3* cKO SPG1, SPG2 and SPG4 cells (Fig. S6). The concerted activity of co-expressed transcription factors and their direct targets (defined as regulons) was used to characterize the germ cell states (Aibar et al., 2017). SCENIC identified 14, 11, 6 and 5 regulons in control SPG1, SPG2, SPG3 and SPG4 clusters, respectively, and 8, 10, 34 and 7 regulons in the corresponding clusters of *Dis3* mutant cells (Fig. 7G, Table S4). We observed that regulon activity grouped concordantly and was specific within each spermatogonial subtype in control and *Dis3* cKO samples (Fig. 7G). The regulon activity changed according to the distinct stages of spermatogonial development in each sample (Fig. 7G). However, the regulons were largely altered within each state between control and *Dis3* cKO samples. Although a few transcription factors overlapped, the number of their direct targets was different (Fig. 7G), suggesting that the expression of regulons was significantly disrupted with loss of DIS3 ribonuclease. Thus, both RNA velocity dynamics and SCENIC results indicate that DIS3 dysfunction alters transcriptional and cellular states, and leads to defects in early spermatogonial development.

## DISCUSSION

**Fig. 8.**
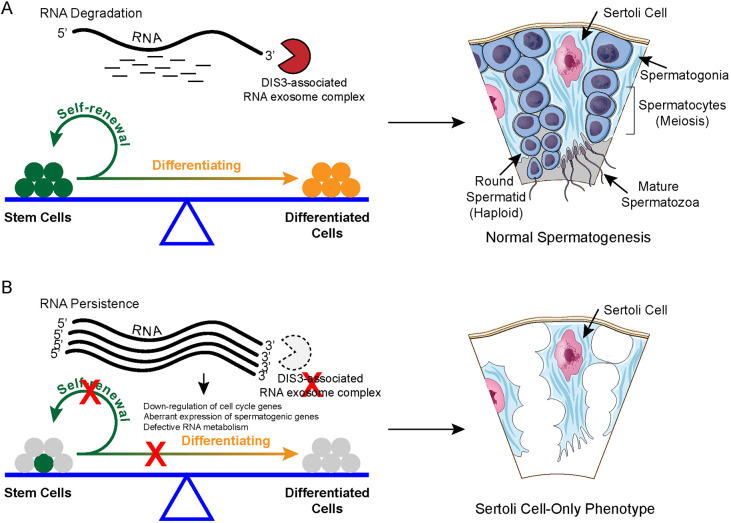
**Model of DIS3 ribonuclease function in early spermatogenic maintenance.** (A) DIS3-mediated RNA degradation is essential for spermatogonial maintenance and thus ensures normal spermatogenesis. (B) DIS3 ribonuclease absence in male germ cells causes downregulation of cell cycle genes, aberrant expression of spermatogenic genes and defective RNA metabolism that impairs spermatogonial maintenance and spermatogenesis, resulting in a Sertoli cell-only phenotype.

SSC self-renewal and differentiation are the basis for generating mature haploid spermatozoa during mammalian spermatogenesis. Abnormalities in SSC homeostasis disrupt spermatogenesis and male fertility. Here, we report that RNA exosome associated DIS3 ribonuclease is required for spermatogonial homeostasis. Conditional disruption of *Dis3* in male germ cells causes downregulation of cell cycle genes, aberrant expression of spermatogenic genes, and defective RNA metabolism that perturbs the maintenance of GFRA1^+^ undifferentiated spermatogonia and impairs transition to progenitor and differentiating spermatogonia (Fig. 8A,B), resulting in severe impairment of germline cell development and subsequent failure of spermatogenesis. Comparative bulk and scRNA-seq data provide evidence that imbalance between RNA synthesis and degradation disrupts RNA abundance and dysregulates gene expression in spermatogonia, which leads to failure of spermatogenic lineage development in mice.

A series of studies in mammalian progenitor cells support a pivotal role for the RNA exosome in maintaining progenitor cells in an undifferentiated state. Deletion of EXOSC9 from human epidermal progenitors leads to decreased self-renewal and precocious differentiation (Mistry et al., 2012). The core exosome components EXOSC8 and EXOSC9, and the catalytic subunit of DIS3 are endogenous suppressors of erythroid cell maturation; disruption of this barricade results in increased mature erythroid cells (McIver et al., 2014). Furthermore, a recent study reported the importance of RNA decay in maintaining human embryonic stem cell (ESC) pluripotency. The results suggest that the RNA exosome complex is required to maintain the properties of human ESCs and depletion of the component EXOSC3 expedites differentiation (Belair et al., 2019). Similarly, our current study demonstrates a pivotal role for DIS3-mediated RNA surveillance pathways in maintaining spermatogonial homeostasis. Although *Dis3* was conditionally ablated in male germ cells by Cre recombinase as early as at E15.5, it seemed that *Dis3* deficiency had no effect on prospermatogonial development, as germ cell number was similar in P2 mutant testes compared with controls. Thus, our study, together with the previous findings, establish the RNA exosome as an important regulator of maintaining progenitor cells in an undifferentiated state.

The exosome-associated DIS3 ribonuclease has a diverse range of targets for nuclear transcript decay, including protein-coding RNAs (mRNAs), transfer RNAs (tRNAs), small nucleolar RNAs (snoRNAs), non-coding RNAs (ncRNAs) and unstable transcripts (including PROMPTs). A comprehensive genome-wide analysis of human DIS3 targets in engineered HEK293 cells documents the essential role of this nuclease in RNA metabolism. Impairment of DIS3 functions leads to global accumulation of PROMPTs, enhancer RNAs (eRNAs), ncRNAs and dysregulation of protein-coding genes (mRNAs) (Szczepińska et al., 2015). Another study reports the immediate impact of rapid deletion of DIS3, EXOSC10 or XRN2 in human cells on nuclear RNA metabolism and transcriptional control. The loss of DIS3 causes substantial accumulation of eRNAs, PROMPTs and products of premature cleavage and polyadenylation (PCPA) (Davidson et al., 2019). Moreover, a recent study documents that both sense and antisense PROMPT reads are significantly increased, and more transcripts are accumulated in mouse *Dis3* cKO (*Zp3-Cre*; *Dis3^F/F^*) oocytes (Wu and Dean, 2023). In our current study, we analyzed the transcriptome profiles in post-natal mouse testes and documented that some DIS3 substrates, including protein coding RNAs, ncRNAs and PROMPTs, are abnormally processed and expressed. PROMPTs are accumulated in both sense and antisense directions, and primarily accumulated in the reverse direction of their downstream genes in *Dis3* cKO testes, consistent with previous findings (Lloret-Llinares et al., 2016; Ntini et al., 2013; Wu and Dean, 2023). Although PROMPTs accumulated in *Dis3* cKO testes, there was no linear relationship between PROMPTs and expression of their downstream genes, similar to an earlier study (Szczepińska et al., 2015). Our study, together with previous findings, suggests a similar role for DIS3 in RNA metabolism across various cell lineages. PROMPTs, as a new class of short, polyadenylated and highly unstable RNAs, were reported more than 10 years ago (Preker et al., 2011, 2008). However, the exact functions of PROMPTs remain unclear. One possibility is that PROMPT transcription may provide reservoirs for RNA polymerase molecules, which can facilitate rapid activation of the downstream gene, and/or alter chromatin structure around the transcriptional start site (Preker et al., 2008). Thus, the potential mechanisms and functions of PROMPTs need to be further investigated. After DIS3 ribonuclease deletion, we noted a significant downregulation of cell cycle and DNA replication genes in RNA-seq and scRNA-seq data, which suggests proliferation defects in *Dis3* cKO spermatogonia. We also observed significant downregulation of transcripts in *Dis3* cKO testes, including some spermatogenic genes, which may account for the defective spermatogonial development and spermatogenesis. The counterintuitive, albeit significant, downregulation of transcripts most likely represents a secondary effect resulting from accumulation of nuclear RNAs, which could sequester factors needed for mRNA biogenesis. Alternatively, the downregulation of spermatogenesis-related genes and proteins may partly be due to spermatogonia composition changes in the mutant testes.

RNA synthesis and degradation maintain a balance for sculpting the cellular transcriptome. Abnormal RNA degradation disrupts this balance and adversely affects gene expression. Our bulk RNA-seq data document that decreased transcripts are enriched in cell cycle, DNA replication, cell division, spermatogenesis and RNA processing. The downregulation of DNA replication and cell cycle have also been observed in EXOSC9 (a key subunit of the exosome complex) depleted human epidermis. EXOSC9 depletion dramatically diminished cell proliferation in the basal layer to less than 5% of control by 27 days post-grafting in immune-deficient mice. Cell cycle analysis of EXOSC9i knockdown cells also demonstrated a loss of proliferation as the cells withdrew from S phase and accumulated in G0/G1 phase (Mistry et al., 2012). Our data, together with previous data, suggest that spermatogonia proliferation defects may directly relate to the downregulation of cell cycle and DNA replication genes. In addition, the disturbed RNA processing causes decreased spermatogenic genes expression (e.g. *Rhox10*, *Sox3*, *Sohlh1* and *Stra8*) and protein expression (e.g. PLZF, LIN28A and KIT), resulting in the impairment of spermatogonial maintenance and spermatogenesis. Our qPCR analysis also verified the downregulation of spermatogenic genes (e.g. *Rhox10*, *Sox3*, *Sohlh1* and *Stra8*), and an immunoblot assay confirmed the reduction of cyclin D1, PLZF, LIN28A and KIT in *Dis3* cKO testes. The alterations of spermatogenic genes in *Dis3* cKO testes may be one of the effects responsible for defective spermatogenesis.

To profile transcriptome signatures on spermatogonia, we re-clustered spermatogonial cells from control and *Dis3* cKO scRNA-seq data. Although we identified four spermatogonial subtypes that followed a continuous differentiation trajectory in both control and *Dis3* cKO spermatogonia, the loss of DIS3 ribonuclease caused significant accumulation of SSCs in SPG1 and a dramatic decrease of differentiated spermatogonia in SPG4. Pathway enrichment analysis showed that DNA replication and cell cycle were significantly downregulated in *Dis3* cKO SPG1, SPG2 and SPG4 cells. This observation further implies that abnormal RNA decay in *Dis3* cKO spermatogonia results in defective spermatogonia proliferation and the impairment of spermatogonial homeostasis. The cellular defect in RNA catabolism also affects RNA splicing and translation processes, which adversely impacts spermatogonial development. However, the dysregulation of RNA splicing and translation observed in bulk and scRNA-seq results is possibly a secondary effect, as DIS3 ribonuclease is responsible for only RNA degradation. Furthermore, RNA velocity dynamics and SCENIC analyses further demonstrate that *Dis3* deficiency alters transcriptional and cellular states that ultimately disrupt early germline cell development.

In summary, our investigations document a causal relationship between the loss of exosome-associated DIS3 ribonuclease-mediated RNA degradation in male germ cells and an adult Sertoli cell-only phenotype that lacks spermatogenic cells. We document that DIS3-associated RNA decay is required for spermatogonial maintenance, normal spermatogenesis and male fertility. Thus, not only do these findings provide new insights into molecular mechanisms of RNA surveillance pathways that regulate spermatogonial biology, they also contribute to new avenues of research to pursue pathogenic mechanisms of the Sertoli cell-only syndrome and male infertility in humans.

## MATERIALS AND METHODS

### Animals

*Ddx4-Cre* transgenic mice were purchased from Jackson Laboratory. All animal studies were performed in accordance with guidelines of the Animal Care and Use Committee of the National Institutes of Health under a Division of Intramural Research, NIDDK approved animal study protocol (K018LCDB24).

### Generation of *Dis3^F/F^* mice

The *Dis3^F/F^* mouse line has previously been established and characterized (Wu and Dean, 2023). The *Dis3* exon map and the strategies for loxP sites insertion are shown in Fig. S1A. The guide RNA (5′-GAGTTGAATGGTTGATTCTT-3′) for the left loxP site and a single-stranded oligo DNA donor containing the left loxP sequence, and the guide RNA (5′-CCTCCCTGGTCTCGTTTGTT-3′) for the right loxP site and a single-stranded oligo DNA donor containing the right loxP sequence were synthesized by Integrated DNA Technologies. Cas9 protein was purchased from Integrated DNA Technologies. Hormonally stimulated B6D2_F1_ (C57LB/6×DBA2) female mice were mated to B6D2_F1_ male mice and zygotes were collected from oviducts at E0.5. sgRNA, Cas9 protein and two DNA oligo donors were mixed and injected into zygotes that were cultured to the two-cell embryo stage and transferred into the oviducts of pseudo-pregnant ICR female mice at E0.5. Genotyping primers for *Dis3^F/F^* mice (right loxP) and *Ddx4* transgenic mice are listed in Table S5.

### Fertility assay

To assess fertility, wild-type female mice were co-caged with either a control or a *Dis3* cKO male mouse for 6 months. The average number of pups per litter was quantified and at least three mating cages were set up for each genotype.

### RNA isolation and quantitative real-time RT-PCR

Total RNA was isolated from mouse tissues using a RNeasy Micro Kit (Qiagen), and cDNA was synthesized with SuperScript III First-Strand Synthesis System (Thermo Fisher Scientific). Quantitative RT-PCR was performed using iTaq Universal SYBR Green Supermix (Bio-Rad) and QuantStudio 6 Flex Real-Time PCR System (Thermo Fisher Scientific). The primers used in this study are listed in Table S6. The relative abundance of each transcript was calculated by the 2^−ΔΔ*Ct*^ normalized to endogenous β-actin expression (Livak and Schmittgen, 2001). Uncropped PCR gels are presented in Fig. S7.

### Immunoblot

Total protein was extracted in 1× LDS sample buffer with 1× NuPAGE Sample Reducing Agent (Thermo Fisher Scientific). Proteins were separated on 4-12% Bis-Tris gels and electrophoretically transferred to PVDF membranes. The membranes were blocked with 5% non-fat milk in Tris-buffered saline containing 0.05% Tween-20 (TBST) at room temperature for 1 h and probed with primary antibodies (Table S7) overnight at 4°C. The membranes were washed three times with TBST and incubated (1 h at room temperature) with secondary antibodies, washed with TBST and developed using SuperSignal West Dura Extended Duration Substrate (Thermo Fisher Scientific). Signals were detected with PXi Touch (SYNGENE) or Hyperfilm ECL (GE Healthcare) according to the manufacturer's instructions. Uncropped blots are presented in Fig. S7.

### Histology analysis

Mouse testes and epididymides were fixed in Bouin's solution overnight at 4°C. Samples were embedded in paraffin wax, sectioned (5 µm) and mounted on slides before staining with periodic acid-Schiff (PAS) and Hematoxylin. Bright-field images were obtained with an inverted microscope (AxioPlan 2; Carl Zeiss).

### Immunofluorescence

Mouse tissues were fixed with 4% paraformaldehyde (PFA) overnight at 4°C, embedded in paraffin wax, sectioned (5 µm) and mounted on slides. After de-waxing, rehydration and antigen retrieval with 0.01% sodium citrate buffer (pH 6.0) (Sigma Aldrich), tissue sections were blocked with blocking buffer containing 3% bovine serum albumin (BSA) and 0.05% Tween-20 at room temperature for 1 h and incubated with primary antibodies (Table S7) overnight at 4°C. Secondary antibodies conjugated to Alexa Fluor (Table S7) were used to detect the antigen and DNA was stained with Hoechst 33342. The cell count was obtained by calculating the marker-positive cells per tubule of the testicular cross-sections. At least 50 tubules were quantified and averaged for each mouse; the experiment was repeated three times.

For whole-mount staining, freshly isolated tissues were fixed in 4% PFA overnight at 4°C, permeabilized in blocking buffer containing 5% donkey serum, 3% BSA, 0.5% Triton X-100 and 0.05% Tween-20 overnight at 4°C and then incubated with primary antibodies (Table S7) for 1-3 days at 4°C. Secondary antibodies conjugated to Alexa Fluor (Table S7) were incubated for an additional 1-3 days at 4°C. After washing in PBS, samples were mounted with PBS on the slides. Fluorescent images were captured by confocal microscopy (LSM 780; Carl Zeiss).

### Isolation of single testicular cells by FACS

Single cells were isolated as described previously (Wang et al., 2019). Briefly, a total of six testes from three different mice per genotype were collected and decapsulated in Hank's Balanced Salt Solution (HBSS, Gibco). Testicular tubules were digested in 15 ml conical tube containing 5 ml (1 mg ml^−1^) of collagenase (Type IV, Sigma Aldrich)/DNase I (Sigma Aldrich) solution in HBSS at 37°C with gentle agitation for 15 min. The dispersed tubules were then digested with 0.25% trypsin/EDTA and DNase I at 37°C with gentle agitation for 10 min. When most of the cells were dispersed, trypsin was neutralized by adding 20% fetal bovine serum (FBS). The cell suspensions were filtered through a pre-wetted 70 µm cell strainer (Corning) and were pelleted by centrifugation at 300 ***g*** for 5 min. The cell pellets were resuspended in HBSS with 15% FBS at a concentration of 1×10^6^ cells ml^−1^.

For FACS analysis, the cells were stained with 1 µg ml^−1^ DAPI to exclude dead cells and stained with DRAQ5 dye (Thermo Fisher Scientific) to quantify DNA content, filtered through a 40 µm cell strainer before loading on a MoFlo Astrios EQ high-speed cell sorter (Beckman Coulter). Flow data analysis was performed using Summit software V6.3.016900 (Beckman Coulter). All testicular cells were sorted into HBSS supplemented with 15% FBS and freshly isolated cells were immediately used for scRNA-seq library preparation.

### RNA-seq library preparation

Total RNA was isolated using a RNeasy Micro Kit and the libraries were constructed using a Universal RNA-Seq Library Preparation Kit (TECAN, 0364) according to the manufacturer's protocol. In brief, double-stranded cDNA was generated with a mixture of random and poly (T) primers, followed by fragmentation of double-stranded cDNA, end repair, adaptor ligation, strand selection, targeted transcript depletion with AnyDeplete, and PCR amplification. The final PCR-amplified libraries were sequenced on an Illumina HiSeq 2500 platform at the NIDDK Genomic Core Facility. Total RNA was isolated from pools of two testes from the same mouse for each library. ‘AnyDeplete’ was used to remove rRNA. Both control and *Dis3* cKO RNA-seq libraries contained five biological replicates.

### scRNA-seq library preparation

scRNA-seq libraries were prepared using Chromium Single Cell 3′ Reagent Kits v3 (10X Genomics) according to the manufacturer's instructions. Briefly, cells obtained from FACS were mixed with a suspension containing barcoded beads and UMI (unique molecular identifier) elements that allow specific tagging of messenger RNA. After partitioning thousands of cells into nanoliter-scale Gel Bead-In-EMulsions (GEMs) and barcoding, full-length barcoded cDNA was then amplified by PCR to generate sufficient mass for library construction. Libraries were constructed by fragmentation, end repair, A-tailing, adaptor ligation and index PCR. After ensuring adequate quality of the cDNA libraries, the samples were sequenced on an Illumina NovaSeq 6000 platform at the NHLBI Genomic Core Facility.

### RNA-seq analysis

Low quality bases and adaptors were trimmed from raw sequence reads with cutadapt v2.7 using parameters -q 20 -a AGATCGGAAGAGC -minimum-length 25. Trimmed reads were mapped to the mouse GRCm39/mm39 genome assembly using HISAT2 v2.1.0 with default parameters (Kim et al., 2019). Aligned reads were counted based on annotation of GENCODE Release M34 using subread featureCounts v1.6.4 with default parameters (Liao et al., 2014), except that the ‘-s’ option was used to specify appropriate strands. Differential expression analysis was carried out in R v3.5.1 version with DESeq2 v1.22.1 (Love et al., 2014). Gene biotype for each transcript was determined according to the annotated ‘gene_biotype’ in the GENCODE M34 GTF file.

### PROMPT analysis

Reads were counted and analyzed from the same BAM files using the same methods as RNA-seq analysis described above, with three modifications:

(1) The annotation file containing PROMPT regions is the unique feature for each of the selected 30,166 transcripts whose PROMPTs do not overlap with any other annotated gene feature. The PROMPT region is −3 kb to −1 bp upstream of the transcription start site.
(2) Because PROMPTs are transcribed bidirectionally with the respect to the downstream genes, we performed sense, antisense and unstranded analysis.
(3) The normalization was estimated based on the library size factor using read counts of all the genes in the GENCODE M34 GTF file for differential gene expression analysis. Gene biotype for each transcript was determined according to the annotated ‘gene_biotype’ in the GENCODE M34 GTF file.

### Correlation analysis between PROMPTs and downstream transcripts

Adjusted *P*-value is false discovery rate (FDR), an arbitrary upper bound on the percentage of false positives of a selected gene list. It was calculated using Benjamini-Hochberg procedure as implemented in DESeq2 software for RNAs-seq analysis. In this method, the *P*-values of gene-wise significant testing are first sorted and ranked. The smallest value is given rank 1, the second is given rank 2, and the largest is given rank N. Then, each *P*-value is multiplied by N and divided by its assigned rank to give the adjusted *P*-values. All the genes that have an adjusted *P*<0.05 in both differential gene expression analysis and PROMPT analysis are included to test whether the log_2_ fold change values are correlated between the accumulation of PROMPTs and their downstream genes using Pearson correlation coefficient analysis.

### scRNA-seq data processing

Raw read processing was carried out using the CellRanger Single-Cell Software Suite (version 3.1, 10X Genomics). In brief, the demultiplexed FASTQ files (28 bp Cell barcode and UMI Read1, 8 bp i7 index, and 100 bp Read2) were generated using the CellRanger *mkfastq* command. The primary data analyses, which included alignment, filtering, barcode counting and UMI quantification for determining gene transcript counts per cell (generated a gene-barcode matrix), quality control, clustering and statistical analysis, were performed using the CellRanger *count* command. Gene positions were annotated using Ensembl build 93 and filtered for biotype (only protein-coding, long intergenic non-coding RNA, antisense, immunoglobulin or T-cell receptor).

### Single-cell transcriptomes to identify cell types

Raw gene expression matrices generated per sample using CellRanger (version 3.1) were imported into R (version 3.6.3) and converted to a Seurat object using the Seurat R package (version 3.1.5) (Butler et al., 2018). Cells that had either fewer than 250 or more than 10,000 expressed genes, or more than 10% UMIs derived from mitochondrial genome were discarded. For the remaining cells, gene expression matrices were normalized with ‘scale.factor=10000 and log1p’ using Seurat *NormalizeData* function. Then, we applied ‘sctransform normalization’ to the data using the Seurat *SCTransform* function with parameter ‘vars.to.regress=c(‘nCount_RNA’, ‘percent.mt’)’, in which gene expression matrices were normalized and scaled, and the highly variably genes (HVGs) were identified. The resulting HVGs were used as features for dimensionality reduction and clustering. The Seurat *RunPCA* was performed to calculate principal components (PCs), which hold the most differences, to separate the cells. The *RunUMAP* function with default setting was then applied to plot the top 35 PCs. The *FindClusters* function with resolution=0.6 parameter was carried out to cluster cells into different groups. Cell cycle scores were calculated using Seurat *CellCycleScoring* function, which shows the cell cycle phase. The canonical marker genes were applied to annotated cell clusters to identify biological cell types.

### Re-clustering of the spermatogonial types

To identify sub-clusters within the spermatogonia-specific cell type, we re-analyzed cells that belonged to spermatogonial cell type. Specifically, cells from the spermatogonial cluster were extracted and germ cell marker genes of *Dazl* and *Ddx4* were used to further confirm cell identity. Cells that did not express specific markers of germ cells were excluded from subsequent analyses. Next, we applied ‘sctransform normalization’ for the data, as described above; cell cycle scores were also calculated using Seurat *CellCycleScoring* function and the gene expression matrices were then further normalized to cell cycle scores. After that, we applied principle component analysis on the selected HVGs for dimensionality reduction. We further performed batch effect correction using Harmony, because a batch effect was observed between control and *Dis3* cKO cells (Korsunsky et al., 2019). A Shared Nearest Neighbor (SNN) graph was constructed from the first 15 Harmony aligned coordinates using the Seurat *FindNeighbors* function. Using the graph-based clustering approach implemented in the *FindClusters* function of the Seurat package, with a conservative resolution of 0.3 and otherwise default parameters, UMAP plots of cells were constructed using the default of the UMAP function *RunUMAP* (Becht et al., 2018).

### Identification of marker genes

To identify marker genes for these cell types, we compared the gene expression values of cells from the cluster of interest with that of cells from the rest of the clusters using the Seurat *FindMarkers* function with default parameter of Wilcoxon rank-sum test. Marker genes were defined based on the following criteria: (1) the average expression value in the cluster of interest was at least 2.5-fold higher than the average expression in the rest of clusters; (2) marker genes should be detectable in at least 10% of the cells in the cluster of interest; and (3) marker genes should have the highest mean expression in the cluster of interest compared with the rest of clusters.

### Gene function analysis

Gene Set Enrichment Analysis (GSEA, version 4.3) was used to complete GO term and pathway enrichment analysis with the Molecular Signatures Database (MSigDB, version 7.1) C5 GO gene sets and C2 curated gene sets (Canonical pathways), respectively.

### Pseudotime analysis

Monocle 3 was used to perform pseudotime analysis. Seurat object for spermatogonial types was converted to Monocle object, and metadata and UMAP embedding were also passed to Monocle object. Data were processed using the ‘preprocess_cds’ function. Cells were clustered using the ‘cluster_cells’ function with cluster_method=‘leiden’. The trajectory graph was learned with the ‘learn_graph’ function and cells were ordered in pseudotime with the ‘order_cells’ function. The pseudotime trajectory plot was generated for the spermatogonial cells.

### RNA velocity analysis

We ran *velocyto* on Cell Ranger using the velocyto R package (version 0.6). ‘Seurat’ and ‘SeuratWrappers’ (version 0.2) were used to process *velocyto* output data, and RNA velocity was estimated using *velocyto.R* function ‘gene.relative.velocity.estimates’. TRNA velocities were then visualized on existing embedding UMAP. RNA velocity analysis was performed in control and *Dis3* cKO spermatogonia separately.

### Gene regulatory network analysis by SCENIC

Investigation of gene regulatory networks was performed using SCENIC in R (Aibar et al., 2017) on control and *Dis3* cKO spermatogonia separately. SCENIC explores gene regulatory networks by identifying transcription factor co-expressed modules with significant motif enrichment. The normalized expression matrix and cell type information generated from Seurat was used as SCENIC input (Van de Sande et al., 2020). Analysis was performed using the mm9 mc9nr motif collection with a window of 10 kb around the transcription start site for running RcisTarget. Heatmaps of gene regulons were plotted using the pheatmap function in R.

### Statistical analysis

Data are mean±s.d. Statistical analyses were performed using GraphPad Prism. The differences between two groups were compared using a two-tailed Student's *t*-test. The significance was defined as ns (no significance), *P<0.05, **P<0.01, ***P<0.001 and ****P<0.0001.

## Supplementary Material

10.1242/develop.202579_sup1 Supplementary information

Table S1.

Table S2.

Table S3.

Table S4.
