# Supplementary material for: DIS3 ribonuclease is essential for spermatogenesis and male fertility in mice
Source: Development. 2024 Jul 2;151(13):dev202579. doi: 10.1242/dev.202579 (PMC11266750; doi:10.1242/dev.202579)
Supplement: Supplementary information [file develop-151-202579-s1.pdf]

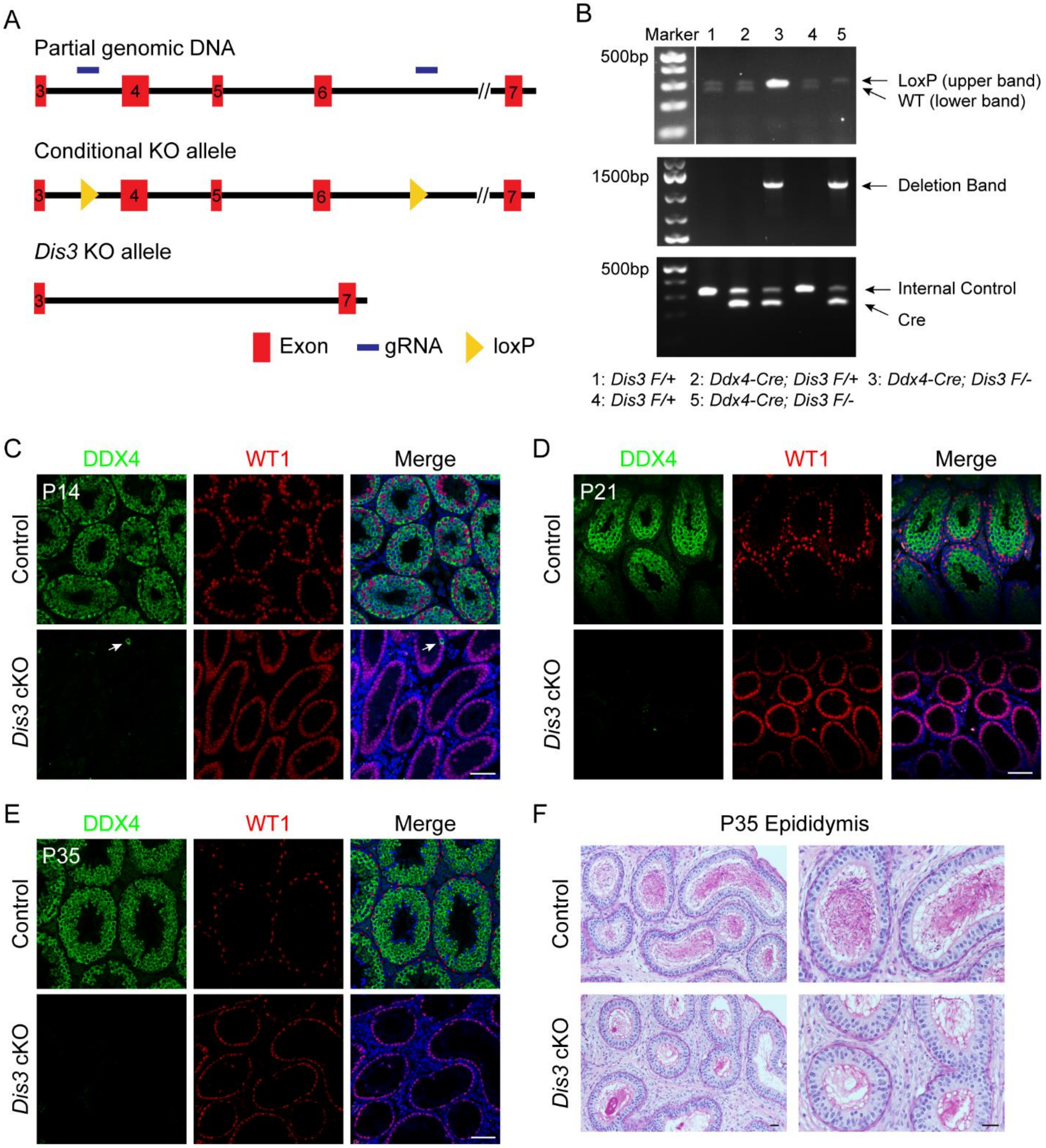

Fig. S1. *Dis3* is required for maintenance of early spermatogenic lineage. (A) Exon map of the mouse *Dis3* locus based on Ensembl (ENSMUSG00000033166). Two guide RNAs (gRNAs) were designed and two loxP sites were inserted as shown in the map. Exons 4 to 6 were deleted after crossing with a *Ddx4-cre* transgenic mouse line, leading to a frame shift mutation of *Dis3* gene. (B) PCR genotyping strategies to detect right loxP band (34 bp longer than wildtype band),

Development • Supplementary information

deletion band (only deleted allele can be amplified) and *cre* band. The genotypes of the five mice were shown. (C) Co-immunostaining of DDX4 (left) or WT1 (middle) and merged with Hoechst 33342 to stain DNA (right) in P14 control and *Dis3* cKO testes. Arrow, germ cell. Scale bar, 50 µm. (D) Same as (C), but in P21 control and *Dis3* cKO testes. (E) Same as (C), but in P35 control and *Dis3* cKO testes. (F) PAS staining of cauda epididymides from P35 control and *Dis3* cKO mice. Scale bar, 50 µm. Representative of n=3 (C-F) independent biological replicates with similar results per condition.

Development • Supplementary information

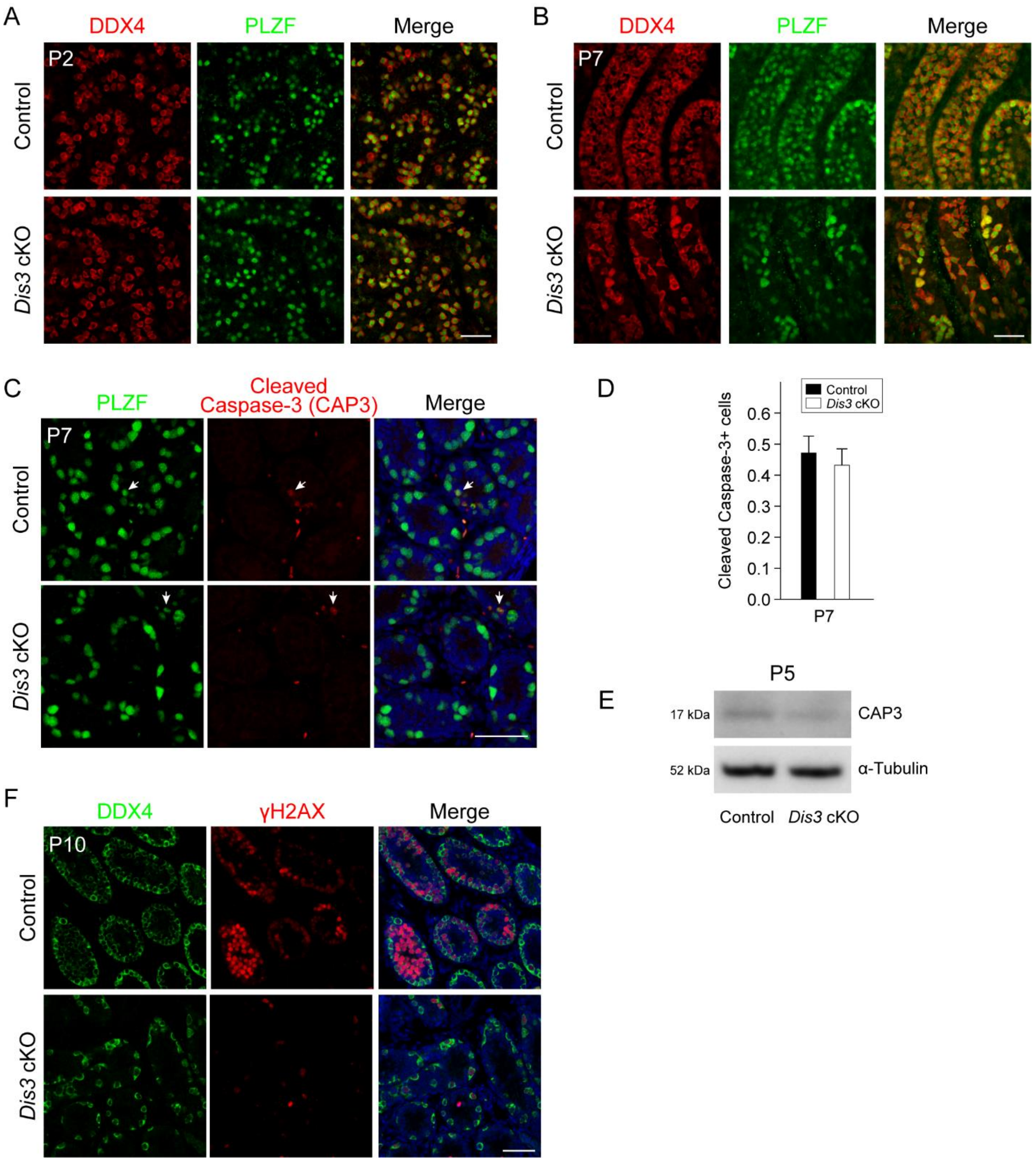

Fig. S2. *Dis3* is essential for spermatogonial maintenance. (A) Immunofluorescence of whole-mount testes from P2 control and *Dis3* cKO mice after staining with antibodies to DDX4 (left) or PLZF (middle) and merged (right). Scale bar, 50 µm. (B) Same as (A), but from P7 control and *Dis3* cKO mice. (C) Immunofluorescence staining of P7 control and *Dis3* cKO testicular sections

Development • Supplementary information

with antibodies to PLZF (left) or cleaved caspase-3 (middle) and merged with Hoechst 33342 to stain DNA (right). Arrows, both PLZF- and cleaved caspase-3-positive cells. Scale bar, 50 µm. (D) Statistical analysis of cleaved caspase-3-positive cells per tubule. Mean ± s.d. (E) Immunoblot of cleaved caspase-3 protein in P5 control and *Dis3* cKO testes using α-tubulin, as also shown in Fig. 2C, as a load control. (F) Co-immunostaining of DDX4 and γ-H2AX in P10 testicular sections from control and *Dis3* cKO mice. The DNA was stained with Hoechst 33342. Scale bar, 50 µm. Representative of n=3 (A-C), (E) and (F) independent biological replicates with similar results per condition. n=3 (D) biologically independent testes from three different animals were analyzed.

Development • Supplementary information

A

Gene Ontology (upregulated)

GO enrichment	Count	P value
Regulation of transcription from RNA Polymerase II promoter	148	3.2E-21
Regulation of transcription	375	6.7E-21
Tube development	85	1.2E-20
Positive regulation of RNA metabolic process	109	2.1E-18
Cell adhesion	129	6.3E-17
Actin filament-based process	59	2.5E-15
Phosphate metabolic process	170	7.9E-15
Heart development	66	4.1E-14
Cell projection organization	83	4.8E-14
Phosphorylation	145	1.0E-13
Tissue morphogenesis	68	1.0E-13
Morphogenesis of an epithelium	55	2.8E-13
Vasculature development	69	4.2E-13
Blood vessel development	67	1.2E-12
Cytoskeleton organization	79	1.0E-11
Epithelium development	66	5.1E-10
Urogenital system development	41	2.3E-8

B

KEGG Pathway (upregulated)

Enrichment terms	Count	P value
Focal adhesion	64	6.7E-17
Pathways in cancer	78	1.3E-12
Adherens junction	30	1.8E-10
Axon guidance	41	2.0E-10
ECM-receptor interaction	30	2.1E-9
Dilated cardiomyopathy	27	1.7E-6
Vascular smooth muscle contraction	31	4.5E-6
GnRH signaling pathway	27	5.0E-6
Regulation of actin cytoskeleton	45	1.2E-5
Wnt signaling pathway	34	2.4E-5
MAPK signaling pathway	51	2.5E-5
Gap junction	23	5.8E-5
Calcium signaling pathway	39	7.5E-5
Endocytosis	37	1.1E-3
Notch signaling pathway	14	1.6E-3
Insulin signaling pathway	24	1.8E-2
TGF-beta signaling pathway	16	3.8E-2

C

Gene Ontology (downregulated)

GO enrichment	Count	P value
Translation	118	1.2E-29
rRNA processing	58	1.1E-25
Cell cycle	124	6.6E-16
DNA replication	39	2.8E-11
Cell division	78	5.5E-11
Ribosome biogenesis	32	6.0E-11
Meiotic cell cycle	33	8.1E-11
Spermatogenesis	81	2.5E-10
piRNA metabolic process	16	9.7E-9
RNA splicing	53	1.4E-8
mRNA processing	64	2.4E-8
tRNA processing	24	2.9E-6
RNA processing	21	1.0E-5
Nucleosome assembly	24	9.9E-5
Synapsis	11	3.0E-4
Gene silencing by RNA	15	3.3E-4
Male meiosis	10	8.2E-4
snRNA processing	6	5.2E-3

D

KEGG Pathway (downregulated)

Enrichment terms	Count	P value
Ribosome	66	1.9E-26
Proteasome	28	1.0E-15
Metabolic pathways	209	4.3E-12
RNA transport	50	2.9E-11
Cell cycle	37	1.0E-8
Parkinson's disease	41	1.9E-8
DNA replication	17	1.4E-7
Huntington's disease	47	2.0E-7
Spliceosome	33	6.6E-6
Alzheimer's disease	40	6.7E-6
mRNA surveillance pathway	23	3.8E-4
Protein processing in endoplasmic reticulum	31	3.0E-3
RNA degradation	16	2.5E-2

E

		PROMPTs:sense		PROMPTs:antisense			PROMPTs:unstranded	
Biotype	PROMPTs ref	Up	Down	Up	(Up%)	Down	Up	Down
lncRNA	4,597	45	0	168	3.65	2	193	1
miRNA	516	5	0	17	3.29	2	20	0
processed pseudogene	6,327	33	6	81	1.28	7	104	16
protein coding	13,269	179	16	2,388	18.00	13	2,162	23
rRNA	125	1	0	3	2.40	0	4	0
snoRNA	550	1	0	8	1.45	1	7	0
snRNA	679	9	0	16	2.36	1	19	0
TEC	739	6	0	21	2.84	1	21	1
transcribed unprocessed pseudogene	162	1	0	4	2.47	0	4	0
unprocessed pseudogene	2,201	4	1	16	0.73	0	17	1
IG_V_gene	196	0	0	1	0.51	0	0	0
IG_V_pseudogene	137	0	0	1	0.73	2	3	0
misc RNA	208	0	0	9	4.33	0	10	0
pseudogene	20	0	0	1	5.00	0	1	0
TR_V_gene	105	0	0	1	0.95	0	0	0
transcribed processed pseudogene	143	0	0	5	3.50	0	7	0
transcribed unitary pseudogene	14	0	0	2	14.29	0	2	0
Total		284	23	2,742		29	2,574	42

Fig. S3. RNA-seq analysis of P4 control and *Dis3* cKO testes. (A) GO enrichment analysis of up-regulated transcripts in P4 *Dis3* cKO testes. GO terms were obtained by DAVID analysis. P value was obtained from the data by DAVID analysis. (B) KEGG pathway analysis of up-regulated genes in *Dis3* mutant testes. The enriched terms were obtained by DAVID analysis. P value was obtained from the data by DAVID analysis. (C) Same as (A), but for down-regulated transcripts. (D) Same as (B), but for down-regulated genes. (E) An overview of the gene biotypes for these differentially expressed PROMPTs-associated transcripts shown in Figure 5E.

Development • Supplementary information

A

Cell Ranger report of the sequencing data

Samples	Number of cells	Number of reads	Total genes	Mean reads/cell	Median genes/cell	Median UMI/cell
Control	17,242	869,712,029	24,648	50,441	3,484	10,240
Dis3 cKO	12,794	1,093,390,982	24,956	85,461	4,132	13,989

Summary for each sample (cleaned data)

Samples	Number of cells	Number of UMI	Total genes	Mean genes/cell	Median genes/cell	Mean UMI/cell	Median UMI/cell
Control	16,429	263,340,632	22,552	3,830	3,555	16,029	10,537
Dis3 cKO	12,015	216,887,266	22,858	4,205	4,209	18,051	14,400

B

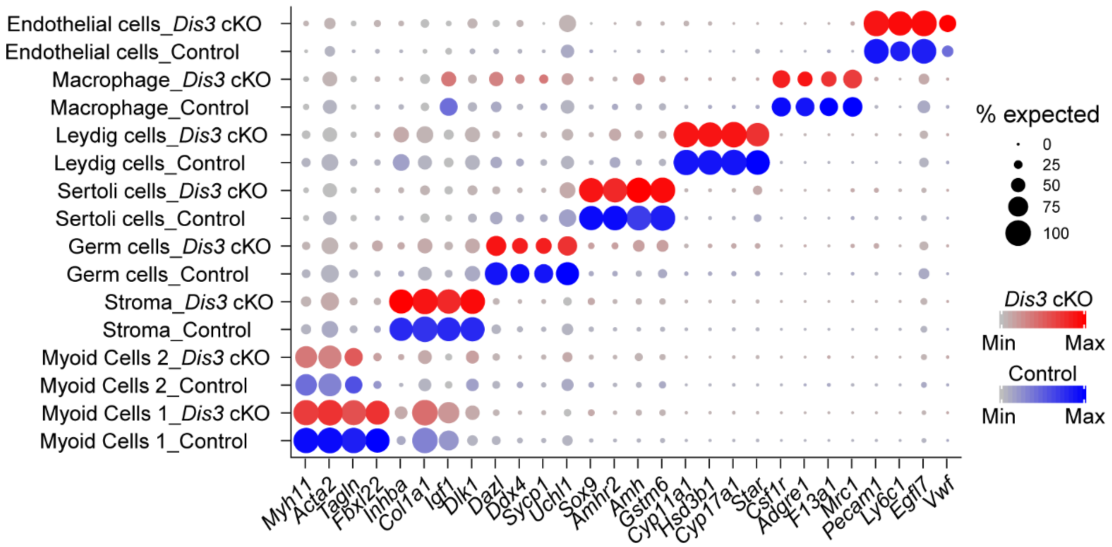

C

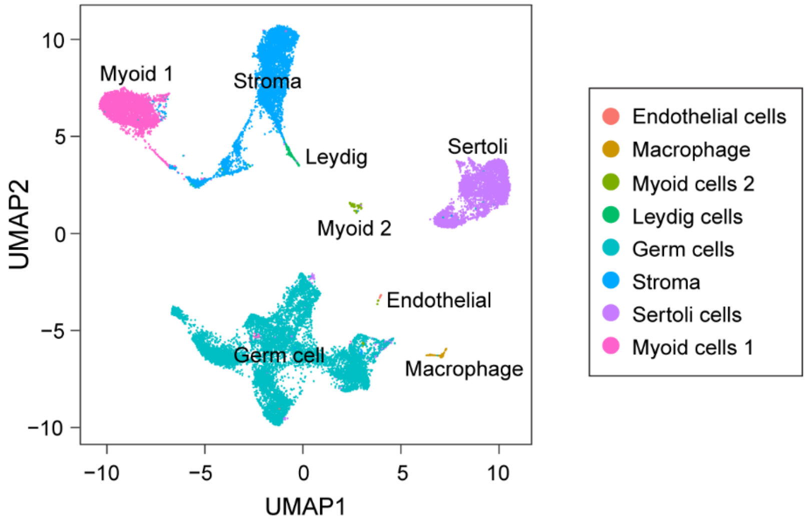

Fig. S4. scRNA-seq profiles and cell type identification of P6 control and *Dis3* cKO testicular cells. (A) scRNA-seq data metrics before and after filtering. (B) Dot plot for the expression of selected marker genes across all identified cell types. (C) UMAP plot of identified cell clusters from P6 control and *Dis3* cKO testicular cells. Each dot represents a single cell and cell types are indicated by colors.

Development • Supplementary information

A

Summary for spermatogonia (cleaned data)

Samples	Number of cells	Number of UMI	Total genes	Mean genes/cell	Median genes/cell	Mean UMI/cell	Median UMI/cell
SPG1	1,519	61,164,503	21,943	6,838	6,824	40,266	38,544
SPG2	1,151	41,760,233	21,565	6,473	6,600	36,282	35,522
SPG3	874	28,484,713	20,529	5,472	6,730	32,591	37,021
SPG4	1,383	45,924,795	20,875	6,262	6,264	33,231	31,892

B

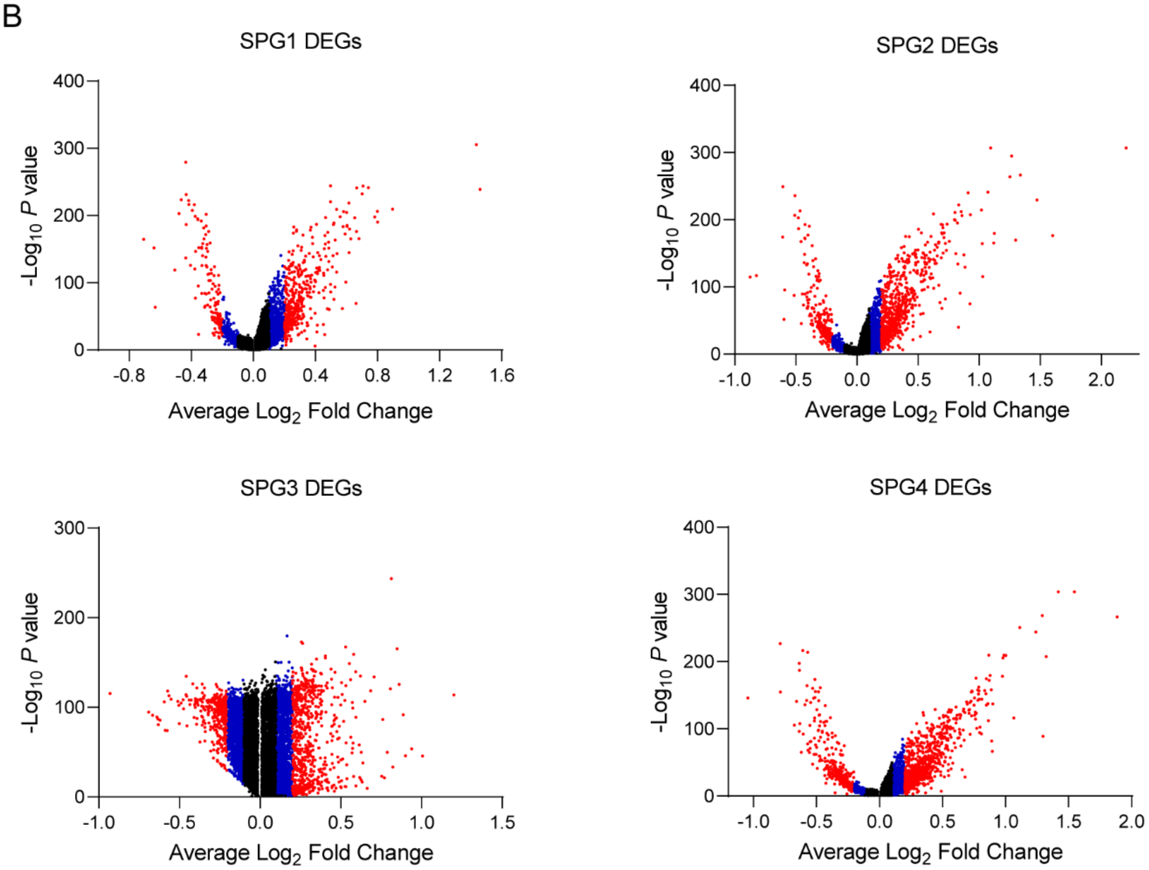

C

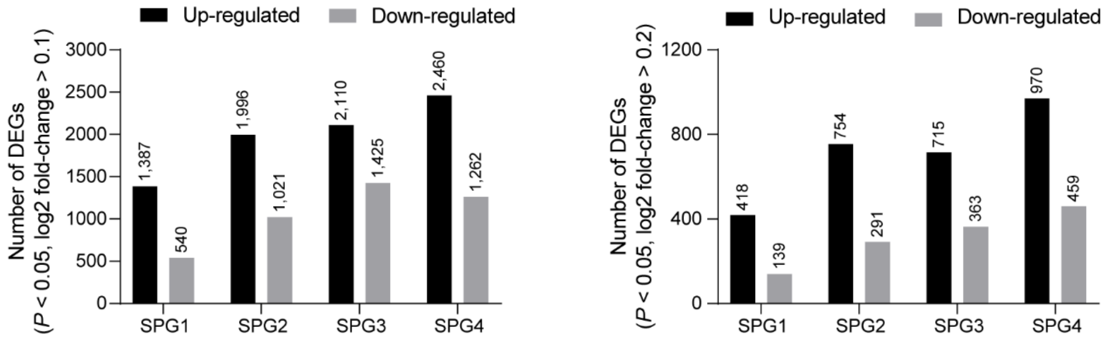

Fig. S5. Differential gene expression analyses in different spermatogonial subtypes. (A) Sequencing metrics of cleaned data for spermatogonia. (B) Volcano plots showing differentially expressed genes (DEGs) of each spermatogonial subtype. Blue points have a log_2 fold change >0.1, red points have a log_2 fold change >0.2. (C) The number of DEGs in each spermatogonial subtype using a cutoff of P <0.05 and log2 fold-change >0.1 (left panel) and P <0.05 and log2 fold-change >0.2 (right panel) between control and *Dis3* cKO testes.

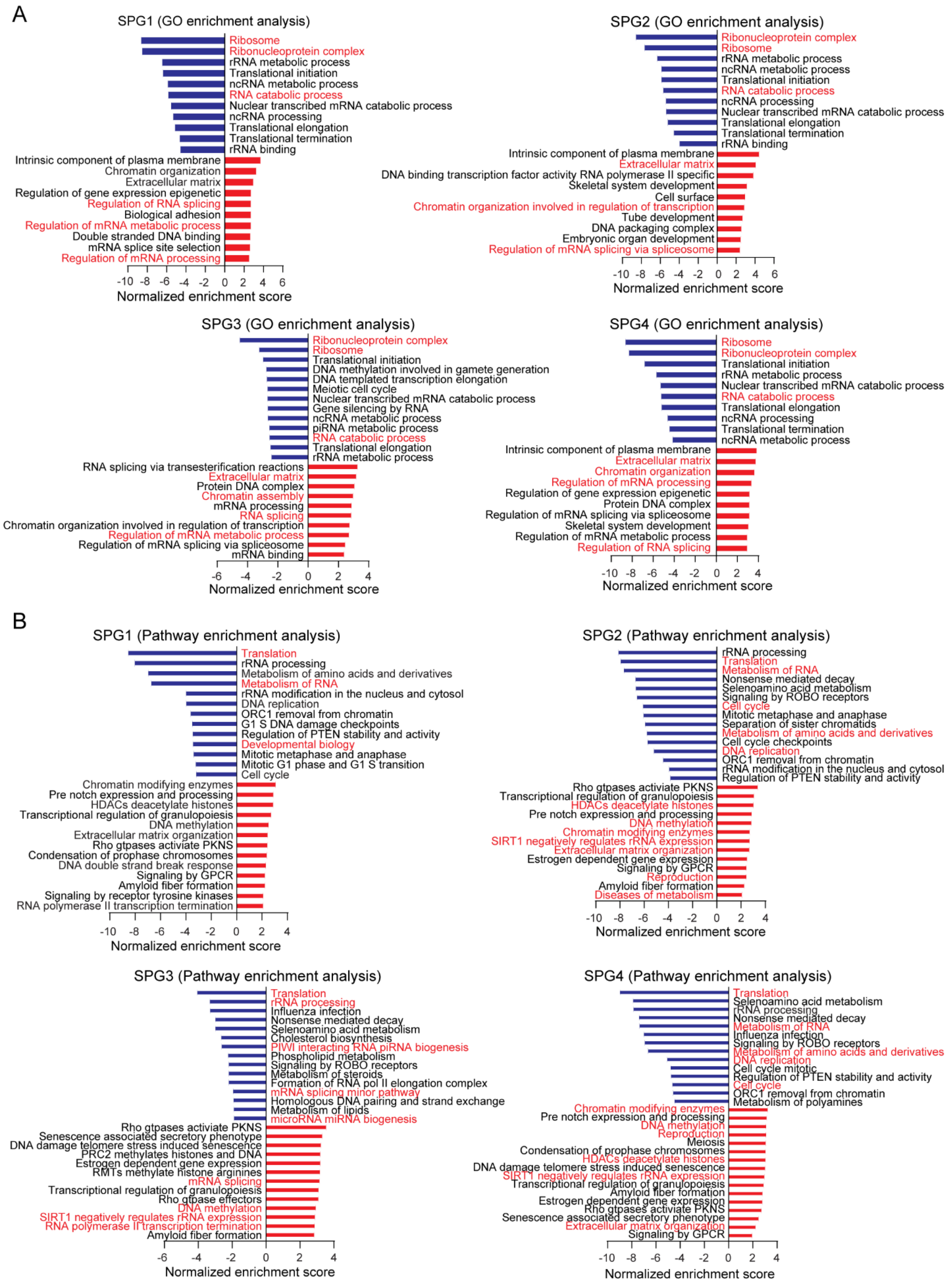

Development • Supplementary information

Fig. S6. GO and pathway enrichment analyses in different spermatogonial subtypes. (A) GO enrichment analysis of biological process categories of *Dis3* cKO spermatogonia compared with control spermatogonia in SPG1, SPG2, SPG3 and SPG4 subtype by using Gene Set Enrichment Analysis (GSEA) software and Molecular Signatures Database (MSigDB) C5 GO gene sets. (B) Pathway enrichment analysis of *Dis3* cKO spermatogonia compared with control spermatogonia in SPG1, SPG2, SPG3 and SPG4 cluster by using GSEA software and the MSigDB C2 curated gene sets.

Development • Supplementary information

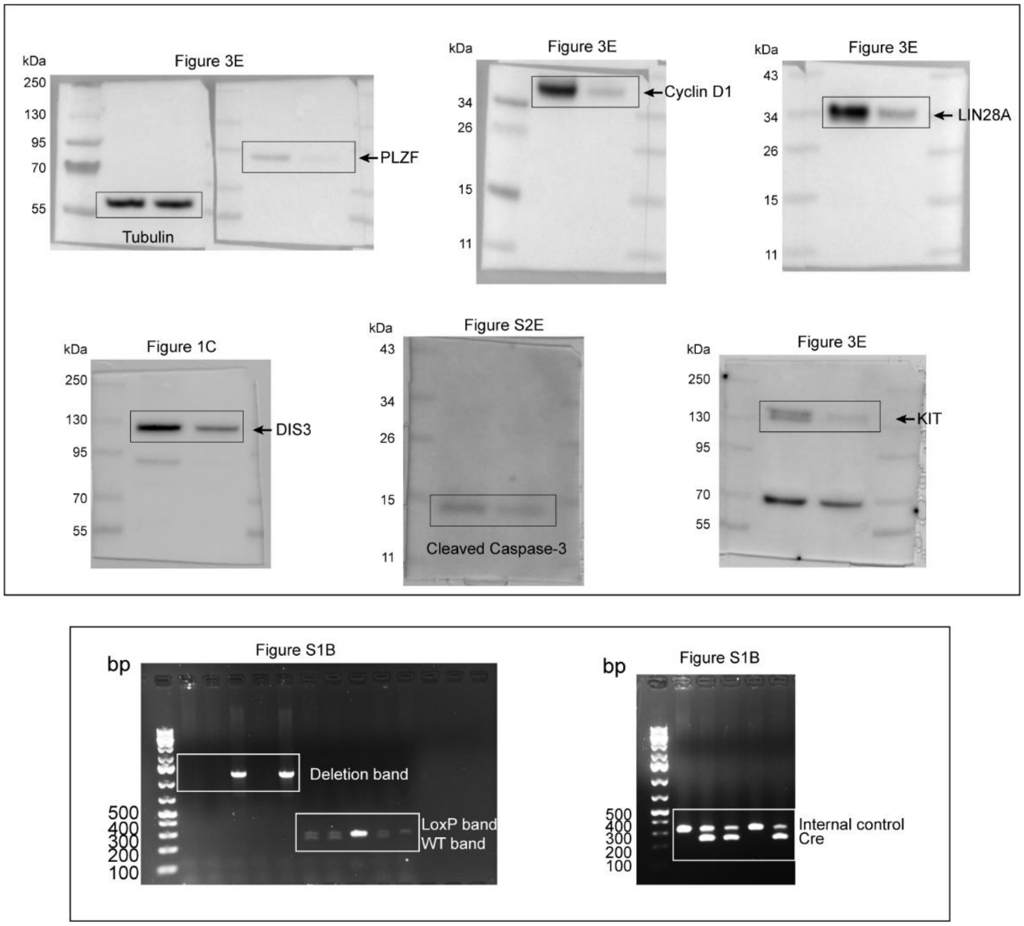

Fig. S7. Uncropped images of PCR gels and immunoblots. Rectangles indicate areas that were cropped for use in figures.

Table S1.

Available for download at
https://journals.biologists.com/dev/article-lookup/doi/10.1242/dev.202579#supplementary-data

Table S2.

Available for download at
https://journals.biologists.com/dev/article-lookup/doi/10.1242/dev.202579#supplementary-data

Table S3.

Available for download at
https://journals.biologists.com/dev/article-lookup/doi/10.1242/dev.202579#supplementary-data

Table S4.

Available for download at
https://journals.biologists.com/dev/article-lookup/doi/10.1242/dev.202579#supplementary-data

Table S5. Genotyping primers for *Dis3* cKO and *Ddx4-Cre* mice

Gene	Direction	Primer (5'-3')
Dis3 LoxP	F[1]	GTAACATTGGAGCATCTACAG
	R	CTCATGGTCATGTTAGATACTAT
Dis3 Deletion	F	ACCACAGGGCTACCTAAATTTAACA
	R	CCTTCCCATCTCAAGCAGAG
Ddx4-cre (Internal control)	F	CAAATGTTGCTTGTCTGGTG
	R	GTCAGTCGAGTGCACAGTTT
Ddx4-cre (Transgene)	F	CACGTGCAGCCGTTTAAGCCGCGT
	R	TTCCCATTCTAAACAACACCCTGAA

[1]F, forward; R, reverse

Table S6. Primers used for RT-PCR

Gene	Direction	Primer (5'-3')
Dis3	F[1]	GCTGCACCAGATTGATGTCCT
	R	CTGATTCGCTTGTAGATGGGG
Plzf	F	CTGGGACTTTGTGCGATGTG
	R	CGGTGGAAGAGGATCTCAAACA
Dmrt1	F	GACCCCGCCTACTACAGCA
	R	GTCTGAGCAGGCACGTAAGG
Pramef12	F	TACAGCTCGCAATGCAAAGC
	R	CCTCAGGATGTTAAGTCGTTTGT
Taf4b	F	TTGCAGCTATTGGACCAAGGA
	R	GTGGCTGTTAGGCTGGAAGT
Nanos3	F	GAGTCCCGTGCCATCTATCAG
	R	GCTGGTGAGTGGGCAAAAG
Rhox10	F	GCCCAATGTCGCTTTGGAAG
	R	AGCTGTAGGTTTGCGTGTATTT
Sox3	F	GAACGCATCAGGTGAGAGAAG
	R	GTCGGAGTGGTGCTCAGG
Sohlh1	F	CGGGCCAATGAGGATTACAGA
	R	TCCTGCGTTCTCTCTCGCT
Stra8	F	CAAAAGCCTTGGCTGTGTTA
	R	AAAGGTCTCCAGGCACTTCA
Sycp3	F	AGCCAGTAACCAGAAAATTGAGC
	R	CCACTGCTGCAACACATTCATA
Tex19.1	F	GGGGATTGGGGTCCCTTTATG
	R	GGGAAAAAGCCCATCAAGTCTC
Mael	F	CCTCCCTTGTGAAATTGGCTG
	R	AATGGAATCGAAATCCTCGTGG
Hormad1	F	GGCTCCTAGCTGTTTCAGTATCT
	R	TTGTCCCATAAGCACGTTCTG
Piwil1	F	AGACCTCATTGGAAGGTGTCA
	R	TGTTCCCCATTCCGAGTCTGA
β-actin	F	GGCTGTATTCCCCTCCATCG
	R	CCAGTTGGTAACAATGCCATGT

[1]F, forward; R, reverse

Development • Supplementary information

Table S7. Antibodies used for immunohistochemistry and immunoblots

Antibody	Company	Identifier	Immuno-blot	Immunohisto-chemistry
Rabbit anti-DIS3	Abcam	Cat# ab223767	1:500	1:200
Mouse anti-DDX4	Abcam	Cat# ab27591; RRID: AB_11139638		1:200
Rabbit anti-WT1	Abcam	Cat# ab89901; RRID: AB_2043201		1:200
Rabbit anti-cyclin D1	Abcam	Cat# ab134175; RRID: AB_2750906	1:1000	1:200
Goat anti-PLZF	R&D Systems	Cat# AF2944; RRID: AB_2218943	1:200	1:200
Goat anti-KIT	R&D Systems	Cat# AF1356; RRID: AB_354750	1:200	1:200
Rabbit anti-cleaved caspase-3	Cell Signaling Technology	Cat# 9661; RRID: AB_2341188	1:500	1:200
Rabbit anti-phospho-histone H2A.X (Ser139)	Cell Signaling Technology	Cat# 9718; RRID: AB_2118009		1:200
Rabbit anti-LIN28A	Cell Signaling Technology	Cat# 8641S; RRID: AB_10997528	1:1000	
Mouse anti-alpha tubulin	Thermo Fisher Scientific	Cat# 62204; RRID: AB_1965960	1:1000	
Donkey anti-mouse IgG, Alexa Fluor 488	Thermo Fisher Scientific	Cat# A-21202		1:200
Donkey anti-rabbit IgG, Alexa Fluor 594	Thermo Fisher Scientific	Cat# A-21207		1:200
Donkey anti-goat IgG, Alexa Fluor 488	Thermo Fisher Scientific	Cat# A-11055		1:200
Donkey anti-mouse IgG, Alexa Fluor 594	Thermo Fisher Scientific	Cat# A-21203		1:200
Goat anti-mouse IgG, HRP	Thermo Fisher Scientific	Cat# 62-6520	1:5000	
Goat anti-rabbit IgG, HRP	Thermo Fisher Scientific	Cat# 31460	1:5000	
Donkey anti-goat IgG, HRP	Thermo Fisher Scientific	Cat# PA1-28664	1:5000	

Development • Supplementary information
